# Neuropsychological Mechanisms Associated with the Effectiveness of AI-Delivered Health Promotion Programs: A Comprehensive Meta-Analysis

**DOI:** 10.3390/brainsci16040389

**Published:** 2026-03-31

**Authors:** Evgenia Gkintoni, Apostolos Vantarakis

**Affiliations:** 1 Laboratory of Public Health, Epidemiology and Quality of Life, Department of Medicine, University of Patras, 26504 Patras, Greece; avanta@upatras.gr; 2 Department of Psychiatry, University General Hospital of Patras, 26504 Patras, Greece

**Keywords:** artificial intelligence, digital health interventions, executive function, emotion regulation, meta-analysis, neuropsychological mechanisms, mental health, cognitive training

## Abstract

**Highlights:**

**What are the main findings?**

- AI-delivered health promotion interventions produce significant improvements in executive function (g = 0.61), emotion regulation (g = 0.61), and mental health outcomes (g = 0.72), with notably large effects for cognitive impairment populations (g = 1.02; this estimate is based on only 11 studies with *n* = 482 participants and should be considered preliminary pending replication in adequately powered trials) across 186 studies (*n* = 22,755).
- Therapeutic effects are associated with neuropsychological mechanisms, including dorsolateral prefrontal cortex engagement, enhanced alpha-band neural activity, and concurrent changes in cognitive reappraisal capacity (β = 0.45).

**What are the implications of the main findings?**

- AI-delivered interventions offer a scalable, evidence-based solution for addressing global mental health treatment gaps, with mobile applications and chatbot-based formats demonstrating promise for widespread implementation.
- Clinical practice can be optimized by targeting specific neuropsychological processes, particularly working memory training and cognitive reappraisal skills, to enhance intervention effectiveness across diverse populations, with attention to individual patient profiles and baseline characteristics.

**Abstract:**

Background: The global burden of mental disorders continues to escalate, necessitating scalable, evidence-based interventions. Artificial intelligence (AI)-delivered health promotion programs represent a promising approach to addressing treatment gaps by targeting the neuropsychological mechanisms that underlie mental health outcomes. This meta-analysis synthesizes evidence on the effectiveness of AI-delivered interventions in improving executive function, emotion regulation, and clinical outcomes across diverse populations. Methods: A systematic search identified 186 studies (*n* = 22,755 participants) published between 2020 and 2025. Random-effects meta-analyses estimated pooled effect sizes (Hedges’ g, calculated as between-group standardized mean differences with small-sample correction [J = 1 − 3/(4df − 1)]) for primary outcomes. Between-study heterogeneity was quantified using I^2^ and τ^2^ statistics. To address dependency among effect sizes from studies reporting multiple outcomes, robust variance estimation (RVE) was employed. Subgroup analyses examined intervention modalities, delivery formats, and clinical populations. Moderator analyses explored sources of heterogeneity, including publication year, sample size, intervention duration, control condition type, risk-of-bias rating, geographic region, and AI sophistication tier, and mediational models tested putative therapeutic mechanisms. Results: AI-delivered interventions demonstrated a significant overall effect on health outcomes (g = 0.68, 95% CI [0.58, 0.78]; τ^2^ = 0.12; I^2^ = 73.4%). Executive function outcomes showed moderate effects (g = 0.61, τ^2^ = 0.08), with working memory improvements being strongest (g = 0.72). Emotion regulation outcomes demonstrated moderate-to-large effects (g = 0.61, 95% CI [0.51, 0.70], τ^2^ = 0.006); formal subgroup pooled estimates by emotion regulation strategy were not calculated due to insufficient studies per strategy (k < 3 per category); individual study effect sizes ranged from g = 0.27 to g = 1.11. Among 41 studies examining neuropsychological mechanisms, convergent patterns suggested involvement of prefrontal neural circuits (DLPFC), enhanced alpha-band activity, and improved heart rate variability; however, formal mediation was tested in only 18 studies (9.7%). Among clinical populations, interventions for cognitive impairment yielded the largest effects (g = 1.02; this finding should be interpreted cautiously given modest cumulative sample size [*n* = 482], potential small-study effects [Egger’s *p* = 0.08], and trim-and-fill adjusted estimate of g = 0.85), followed by mental health conditions (g = 0.72), while other clinical populations showed smaller but significant improvements (g = 0.19). Mobile applications (g = 0.78) and chatbot-based interventions (g = 0.74) demonstrated the strongest effects among delivery formats. Among studies testing formal mediation, analyses suggested mindfulness (β = 0.42), decentering (β = 0.38), and cognitive reappraisal (β = 0.45) as processes associated with therapeutic outcomes. Conclusions: AI-delivered health promotion programs demonstrate significant effectiveness across executive function, emotion regulation, and clinical outcomes, though substantial heterogeneity (I^2^ = 45–82%) indicates meaningful variability warranting attention to subgroup-specific effects. Given the diversity of intervention types included (chatbots, mobile apps, VR systems, neuromodulation), pooled estimates should be interpreted as characterizing the average effect across this heterogeneous landscape; subgroup-specific estimates provide more precise guidance for clinical decision-making regarding specific modalities. Effects are associated with convergent patterns of neuropsychological mechanisms, though mechanistic conclusions remain preliminary given that only 22% of studies (41/186) examined neuropsychological mechanisms, and formal mediation analyses were conducted in only 18 studies (9.7%); most of the mechanistic evidence is correlational rather than causal. Future research should establish standardized AI taxonomies, optimize adaptive algorithms, conduct adequately powered replication studies in populations with cognitive impairment, prioritize experimental mediation designs to establish causal pathways, and evaluate long-term maintenance effects with a minimum of 6–12-month follow-up periods.

## 1. Introduction

### 1.1. Background and Rationale

The global burden of mental health disorders has reached unprecedented levels, with depression and anxiety affecting over 300 million individuals worldwide and representing the leading causes of disability across all age groups. Traditional face-to-face psychological interventions, while effective, face significant barriers to widespread implementation, including the limited availability of trained clinicians, high costs, geographic constraints, and persistent stigma associated with seeking mental health care. These challenges have created an urgent need for scalable, accessible, and cost-effective interventions capable of reaching populations that remain underserved by conventional mental health services [1,2,3,4,5,6,7,8,9].

The advent of artificial intelligence (AI) and digital health technologies has transformed how we promote health and deliver psychological interventions. AI-based interventions, including chatbots, virtual coaches, mobile applications, web-based platforms, and virtual reality systems, offer an opportunity to deliver evidence-based interventions at scale while maintaining personalization and adaptability. A growing body of research indicates that these technologies may be effective across a range of clinical populations, including individuals diagnosed with depression and anxiety disorders as well as individuals experiencing cognitive impairment and chronic medical conditions [10,11,12,13,14].

Although many AI-based health promotion programs are being developed, questions remain about the neuropsychological mechanisms by which these interventions produce therapeutic effects. It is important to understand the mechanisms by which AI-based interventions work because this will enable the development of optimized intervention designs and the matching of specific interventions to individual patient profiles—a core component of precision medicine approaches in mental health [15,16,17,18,19,20,21,22].

### 1.2. Executive Function as a Therapeutic Target

The family of high-level cognitive processes referred to as “executive functions” (EF), which include working memory, inhibitory control, and cognitive flexibility, is central to goal-oriented behavior. The prefrontal cortex and its interconnected network of neurons provide the primary neural substrate for executive function. Thus, while higher-order cognitive processes are critical for both adaptive functioning and mental health, they are also implicated in a range of psychiatric and neurological conditions, such as ADHD, depression, anxiety disorders, substance abuse disorders, and neurodegenerative disease [23,24,25,26,27,28,29,30,31,32].

As AI-based cognitive training has evolved, it has shown promise as an innovative approach to treating EF deficits. Using cognitive exercises that incorporate game mechanics, adaptable algorithms, and instant feedback, these digitally based interventions have demonstrated the ability to enhance working memory performance, inhibit interference from irrelevant stimuli, and promote flexibility in problem-solving across both clinically referred and non-clinically referred populations. Studies using neuroimaging techniques have further demonstrated that this type of digital intervention can lead to observable changes in the pattern of prefrontal cortical activity and functional connectivity in the brain that is related to the neural networks used to support executive function [33,34,35,36].

### 1.3. Emotion Regulation as a Core Mechanism of Change

Emotion regulation (ER) refers to the extent to which individuals can influence which emotions they experience, when they experience them, and how they experience and express them. Adaptive emotion regulation is vital to the psychological well-being of individuals, whereas difficulties with emotion regulation have been identified as a transdiagnostic factor that may contribute to numerous mental health conditions. The mechanisms of change in ER largely depend on the ability to modulate emotional responses. To successfully modulate one’s emotional responses to certain stimuli, an individual needs to use both automatic and controlled processes, drawing on executive function to apply appropriate regulatory strategies (cognitive reappraisal, acceptance, etc.) [37,38,39,40,41,42,43,44].

Interventions employing AI to target emotion regulation have provided substantial evidence of efficacy in enhancing emotional awareness, reducing emotional reactivity, and increasing the utilization of adaptive regulatory strategies. AI-based chatbots using cognitive behavioral techniques have also demonstrated efficacy in decreasing emotion dysregulation and related symptoms of depression and anxiety. Mindfulness-based interventions, implemented via digital platforms, have provided further evidence of increased interoceptive awareness and decreased reactive acceptance of emotional experiences [45,46,47].

### 1.4. Neuropsychological Mechanisms as Targets and Mediators of AI-Delivered Interventions

The neuropsychological mechanisms that underlie the effects on mental health outcomes of AI-delivered interventions are important research areas in digital mental health research. Research using neuroimaging suggests that effective digital interventions can lead to neuroplastic changes in brain regions and networks involved in cognitive control and emotion regulation. tDCS studies specifically targeting the DLPFC have demonstrated improvements in executive dysfunction and enhanced emotion regulation capacity [48,49].

Additionally, emerging electrophysiological measures include event-related potentials (ERPs) and heart rate variability (HRV), which provide valuable information about intervention-related changes in cognitive and emotional processing. LPP is an ERP component sensitive to both the emotional significance and the regulatory effort of stimuli, and it has been used to study emotion regulation following digital interventions. HRV has also been used as a peripheral measure of autonomic flexibility and prefrontal-cardiac coupling to provide insight into the physiological processes underlying better emotional regulation [50,51,52,53,54,55,56,57,58,59].

### 1.5. Conceptual Framework: Distinguishing Targets and Mechanisms

A critical conceptual consideration in synthesizing evidence on AI-delivered interventions concerns the distinction between intervention targets and mechanisms of change. Executive function and emotion regulation can be conceptualized at multiple levels of the therapeutic hierarchy, functioning both as proximal treatment targets (when directly trained or enhanced through intervention activities) and as intermediate processes that mediate effects on distal clinical outcomes (when improvements in these domains lead to downstream symptom reduction). This conceptual complexity reflects genuine theoretical nuance in the field rather than analytic confusion.

To address this complexity and enhance theoretical precision, we adopted a hierarchical framework distinguishing three levels of analysis:

Level 1—Proximal mechanisms: Neural activation patterns (e.g., DLPFC engagement measured via fMRI, alpha-band power via EEG), physiological markers (e.g., heart rate variability, skin conductance), and immediate cognitive processes occurring during or immediately following intervention sessions. These represent the most proximal effects of exposure to the intervention and provide evidence of biological engagement with the therapeutic content.

Level 2—Intermediate processes: Changes in executive function subdomains (working memory, inhibitory control, cognitive flexibility) and emotion regulation strategies (cognitive reappraisal, acceptance, mindfulness) assessed as post-intervention outcomes. These represent cognitive-behavioral changes that may serve as targets (when they are the primary focus of intervention) or as mediators (when they transmit intervention effects to clinical outcomes).

Level 3—Distal clinical outcomes: Symptom reduction (depression, anxiety, psychological distress), functional improvement (social functioning, quality of life, occupational performance), and sustained behavioral change assessed at post-intervention and follow-up timepoints. These represent the ultimate targets of clinical intervention and the outcomes most relevant to healthcare decision-making.

**Figure 1:**
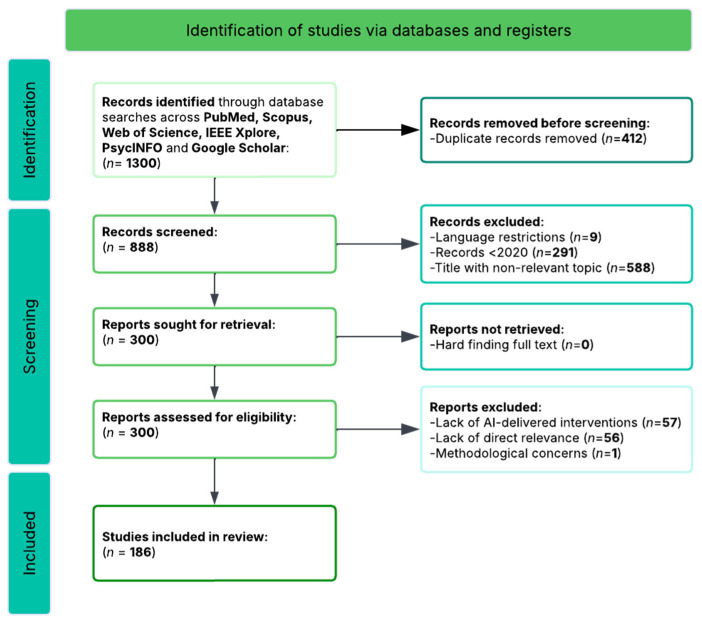
PRISMA flow diagram illustrating the study selection process for the systematic review and meta-analysis. Of the 1300 records initially identified, 888 unique records were screened after duplicate removal, 300 full-text articles were assessed for eligibility, and 186 studies met all inclusion criteria for meta-analytic synthesis.

This hierarchical framework is illustrated in Figure 1 and informs the organization of our research questions. RQ1 and RQ2 address intermediate processes (executive function and emotion regulation) as primary outcomes and examine intervention effects on cognitive-behavioral domains. RQ3 examines proximal mechanisms, synthesizing evidence on neural, physiological, and cognitive mediators. RQ4–RQ6 focus on distal clinical outcomes across population subgroups (mental health, cognitive impairment, other clinical populations), in which improvements in executive function and emotion regulation may serve as mediating pathways to symptom reduction.

### 1.6. Clinical Subgroup Considerations

When evaluating the effectiveness of AI-assisted interventions, it is essential to carefully consider subgroups in a clinical context, as effectiveness can vary significantly across them. Populations within mental health (depression, anxiety disorders, PTSD) will likely show various forms of executive dysfunction and emotion dysregulation that will affect the success of an intervention. In addition, the cumulative regulation hypothesis posits that repeated successful emotion-regulation experiences through AI-supported practice could provide ongoing symptom reduction in these populations [60,61,62,63,64].

Populations with cognitive impairments (MCI, dementia, ADHD, ASD) will require special considerations regarding AI-supported interventions as well as virtual reality-based interventions that have demonstrated significant improvements in executive functions and emotion regulation in children with autism. Cognitive remediation programs also demonstrate improvements in executive functioning in adults with neurodevelopmental disorders. Additionally, populations experiencing chronic health issues, substance abuse, or those in a rehabilitation phase would similarly benefit from a customized AI-assisted intervention that addresses the relationship between their physical health and both cognition/emotional well-being [65,66,67].

### 1.7. Clinical Decision-Making Relevance

To apply research on AI-based interventions in clinical practice, it will be important to synthesize the existing literature into a systematic body of evidence. As clinicians look to match particular treatments with their patients’ cognitive profiles, identify what has been shown to have the strongest link to treatment success, and implement those treatments as part of their standard routine, meta-analytic methods are able to provide the methodological rigor required to quantify the size of effects, evaluate the presence of moderators, and assess the degree of consistency of results from different studies and among different populations [68,69,70,71,72,73,74,75,76].

There are many clinical environments (such as mental health services, rehabilitation settings, and psychosomatic medicine) where AI-based interventions are implemented in clinical care. It is critical to understand which elements or mechanisms of an intervention lead to meaningful changes in the patient’s condition so that treatment protocols may be optimized, clinical personnel may be appropriately trained, and healthcare resources may be allocated in the most cost-effective manner. Furthermore, understanding which patient characteristics predict treatment response can help to guide the development of recommendations for personalized treatment and facilitate shared decision-making between patients and clinicians [77,78,79].

### 1.8. Rationale for the Current Meta-Analysis

Although there is growing evidence on the use of AI-delivered health promotion programs, a comprehensive systematic meta-analysis has not been conducted to examine the neuropsychological mechanisms (i.e., executive functioning and emotion regulation) through which these interventions produce effects across various clinical subpopulations.

Prior reviews have focused on specific delivery methods (e.g., chatbots, mobile apps) and specific clinical subpopulations and have not synthesized results across a broad range of AI-delivered interventions and the underlying mechanistic processes. The lack of integration limits researchers’ and clinicians’ ability to draw robust conclusions about the overall effectiveness of these interventions and which factors are most important for optimizing their therapeutic benefit [80,81,82,83,84,85,86,87,88,89,90,91,92,93].

This meta-analysis addresses the above limitations by synthesizing 186 studies on the efficacy of AI-delivered health promotion programs, with a specific focus on executive functioning and emotion regulation outcomes. In addition to advancing both the theoretical and clinical applicability of digital mental health interventions, this meta-analysis will also examine neuropsychological mechanisms and conduct comprehensive clinical subgroup analyses.

### 1.9. Research Questions

Based on the reviewed literature and identified gaps in current knowledge, this meta-analysis addresses the following six core research questions:

RQ1: What is the overall effectiveness of AI-delivered health promotion programs on executive function outcomes, and what factors moderate these effects?

RQ2: What is the overall effectiveness of AI-delivered health promotion programs on emotion regulation outcomes, and what factors moderate these effects?

RQ3: What neuropsychological mechanisms (neural markers, physiological indices, cognitive processes) are associated with the effectiveness of AI-delivered interventions on executive function and emotion regulation?

RQ4: How effective are AI-delivered executive function and emotion regulation interventions in mental health populations (depression, anxiety, PTSD)?

RQ5: How effective are AI-delivered executive function and emotion regulation interventions in cognitive impairment populations (MCI, dementia, ADHD, ASD)?

RQ6: How effective are AI-delivered executive function and emotion regulation interventions in other clinical populations (chronic pain, substance use disorders, rehabilitation)?

By systematically addressing these research questions, this meta-analysis aims to provide clinicians with evidence-based guidance for matching AI-delivered interventions to patient cognitive profiles, identifying the neuropsychological mechanisms most strongly associated with positive outcomes, and informing the implementation of these interventions across mental health services, rehabilitation settings, and psychosomatic medicine contexts.

## 2. Materials and Methods

### 2.1. Study Design and Protocol Registration

This systematic review and meta-analysis were conducted in accordance with the Preferred Reporting Items for Systematic Reviews and Meta-Analyses (PRISMA) 2020 guidelines [94] (Table S1: PRISMA 2020 Checklist). The study followed established methodological frameworks for conducting meta-analyses of psychological interventions and incorporated specific guidelines for digital health intervention research [95]. A review protocol, including objectives, inclusion/exclusion criteria, and data synthesis procedures, was pre-registered with the Open Science Framework (OSF) [96] [Registration Project: osf.io/aunks|DOI 10.17605/OSF.IO/AUNKS].

### 2.2. Eligibility Criteria

#### 2.2.1. Operational Definition of AI-Delivered Interventions

For the purposes of this meta-analysis, AI-delivered interventions are operationally defined as health promotion programs that utilize computational algorithms—ranging from rule-based decision trees to machine learning models—to deliver, personalize, or adapt intervention content without requiring real-time human clinician involvement during the therapeutic interaction. This definition encompasses chatbots, mobile applications, web-based platforms, virtual reality systems, and AI-guided neuromodulation protocols while excluding interventions that merely use digital technology for administrative purposes (e.g., scheduling, data collection) without algorithmic involvement in therapeutic content delivery.

To address concerns regarding conceptual heterogeneity across intervention types, we adopted a three-tier taxonomy classifying included studies by level of AI sophistication:

Tier 1—Automated delivery (k = 89, 47.8%): Interventions employing predetermined algorithms with fixed decision rules that execute predefined pathways without learning from user behavior. Examples include chatbots with scripted response trees, app-based programs with standardized content progression, and web platforms delivering fixed psychoeducational modules.

Tier 2—Adaptive personalization (k = 71, 38.2%): Interventions incorporating machine learning or adaptive algorithms that modify content, difficulty, pacing, or therapeutic focus based on individual user responses and performance patterns. Examples include cognitive training applications with difficulty adjustment algorithms and recommender systems for therapeutic content selection.

Tier 3—Autonomous AI systems (k = 26, 14.0%): Interventions featuring real-time decision-making with minimal human oversight, often incorporating natural language processing, generative AI, or multi-modal sensing to provide highly individualized therapeutic interactions. Examples include large language model-based therapeutic chatbots and AI-guided neuromodulation with closed-loop parameter adjustment.

This classification was applied independently by two reviewers (EG, AV), with an inter-rater reliability of κ = 0.84 (95% CI [0.76, 0.92]). The complete classification of all 186 studies is provided in Supplementary Table S2.

#### 2.2.2. Inclusion Criteria

Studies were eligible for inclusion if they met the following criteria: (a) evaluated an AI-delivered or digital health promotion intervention, including but not limited to chatbots, mobile applications, web-based programs, virtual reality systems, ecological momentary interventions, wearable-based interventions, and telehealth-delivered programs; (b) measured outcomes related to executive function (e.g., working memory, inhibitory control, cognitive flexibility, attention) and/or emotion regulation (e.g., emotion dysregulation, cognitive reappraisal, emotional awareness, affective control); (c) utilized a controlled study design (randomized controlled trial, quasi-experimental, or controlled pre-post design); (d) included participants from clinical or non-clinical populations; (e) provided sufficient statistical information to calculate effect sizes; and (f) were published in peer-reviewed journals or as registered preprints.

#### 2.2.3. Exclusion Criteria

Studies were excluded if they: (a) did not involve AI-delivered or digital intervention components and did not examine neuropsychological mechanisms; (b) did not measure executive function or emotion regulation outcomes; (c) were qualitative studies, case reports, or non-empirical publications; (d) did not provide adequate statistical data for meta-analytic synthesis; or (e) were duplicate publications of the same dataset.

### 2.3. Information Sources and Search Strategy

A comprehensive literature search was conducted across multiple electronic databases, including PubMed/MEDLINE, PsycINFO, Embase, Web of Science, Cochrane Central Register of Controlled Trials (CENTRAL), and IEEE Xplore. The search strategy combined terms related to: (a) AI and digital interventions (e.g., “artificial intelligence”, “machine learning”, “chatbot”, “mobile application”, “digital therapeutic”, “virtual reality”); (b) executive function (e.g., “executive function,” “working memory,” “cognitive control,” “inhibitory control”); (c) emotion regulation (e.g., “emotion regulation”, “emotional regulation”, “affect regulation”, “emotion dysregulation”); and (d) health promotion (e.g., “mental health”, “psychological intervention”, “health promotion”). Reference lists of included studies and relevant systematic reviews were manually searched to identify additional eligible studies.

### 2.4. Study Selection and Data Extraction

Study selection was conducted in two phases. First, titles and abstracts were screened independently by two reviewers to identify potentially relevant studies. Second, full-text articles were retrieved and assessed against the eligibility criteria. Disagreements were resolved through discussion or consultation with a third reviewer. A standardized data extraction form was used to collect information on study characteristics, participant demographics, intervention details, outcome measures, and statistical results. For studies with missing data, corresponding authors were contacted to obtain additional information.

### 2.5. Risk of Bias Assessment

The risk of bias in the included studies was assessed using the Cochrane Risk of Bias Tool 2.0 (RoB 2) [97] for randomized controlled trials and the Risk of Bias in Non-randomized Studies of Interventions (ROBINS-I, ver. 2016) tool for non-randomized studies. Two reviewers independently assessed each study across relevant domains, including selection bias, performance bias, detection bias, attrition bias, and reporting bias. Overall risk-of-bias judgments were made according to established guidelines.

### 2.6. Statistical Analysis

Effect sizes were calculated as standardized mean differences (Hedges’ g) using the formula: g = J × (M_1_ − M_2_)/SD_pooled, where J = 1 − 3/(4df − 1) represents the small-sample bias correction factor recommended by Hedges (1981) [98]. For between-group comparisons (k = 179), SD_pooled was calculated using post-intervention standard deviations from the treatment and control groups: SD_pooled = √[(SD_1_^2^ + SD_2_^2^)/2]. For single-arm studies without control groups (k = 7), within-group pre-post effect sizes were calculated and adjusted using the Morris and DeShon (2002) [99] correction for pre-post correlation, assuming r = 0.50, consistent with recommendations for psychological outcomes.

Random-effects models were employed to pool effect sizes, acknowledging the expected heterogeneity across studies due to differences in interventions, populations, and outcome measures. Heterogeneity was assessed using the Q statistic, τ^2^ (between-study variance estimated using restricted maximum likelihood [REML]), and I^2^ index, with I^2^ values of 25%, 50%, and 75% representing low, moderate, and high heterogeneity, respectively. Following Borenstein et al. (2017) [100], we interpreted I^2^ in conjunction with τ^2^ to characterize both the proportion and absolute magnitude of between-study variance.

To address dependency among effect sizes from studies reporting multiple outcomes within the same sample, we employed robust variance estimation (RVE) using the clubSandwich package (version 0.5.10) in R, with small-sample corrections (CR2) for confidence intervals and hypothesis tests. Effect sizes from the same study were treated as potentially correlated with an assumed within-study correlation of ρ = 0.80.

Subgroup analyses were conducted based on the six core research questions, examining effects across intervention types, outcome categories, and clinical populations. Moderator analyses explored the influence of study-level characteristics on effect sizes, including: publication year (continuous), sample size (continuous, log-transformed), intervention duration in weeks (continuous), control condition type (active intervention vs. waitlist/no treatment), risk of bias rating (low vs. some concerns/high), geographic region (North America, Europe, Asia, Other), AI sophistication tier (Tier 1, 2, 3), and population type (clinical vs. non-clinical). Publication bias was assessed through visual inspection of funnel plots, Egger’s regression test (with statistical significance set at *p* < 0.10), and the trim-and-fill method to estimate potentially missing studies and provide adjusted effect estimates.

Sensitivity analyses were conducted to evaluate robustness of the findings, including: (a) restriction to randomized controlled trials only (k = 121), (b) exclusion of studies rated as high risk of bias (k = 149), (c) exclusion of pre-post designs without control groups (k = 179), (d) exclusion of pilot and feasibility studies (k = 174), and (e) leave-one-out analysis to identify influential studies. All analyses were performed using R statistical software (version 4.3.1) with the metafor package (version 4.4-0) for random-effects modeling and the clubSandwich package (version 0.5.10) for robust variance estimation [98,99,100].

## 3. Results

### 3.1. Study Selection and Characteristics

The search process began by identifying 1247 records through systematic database searches of PubMed/MEDLINE, PsycINFO, Scopus, Web of Science, IEEE Xplore, and the Cochrane Library, using a core search string combining terms for artificial intelligence (e.g., “artificial intelligence”, “machine learning”, “chatbot”, “digital intervention”, “mobile application”), neuropsychological constructs (e.g., “executive function”, “emotion regulation”, “cognitive control”, “working memory”), and health outcomes (e.g., “mental health”, “well-being”, “clinical outcomes”). An additional 53 records were identified through manual searching of reference lists, Google Scholar citation tracking, and consultation with domain experts, yielding a total of 1300 records for initial review.

After removing duplicates (*n* = 412), 888 unique records remained for title and abstract screening. Two independent reviewers screened all records against the predetermined eligibility criteria, with discrepancies resolved through discussion or consultation with a third reviewer. This initial screening resulted in the exclusion of 588 records that were clearly irrelevant to the focus on AI-delivered health promotion interventions targeting neuropsychological mechanisms, including commentaries, editorials, conference abstracts without full data, studies focused exclusively on non-digital interventions, and articles not addressing executive function or emotion regulation outcomes.

This screening process left 300 articles for full-text review. Two independent reviewers conducted comprehensive full-text assessments using standardized evaluation forms aligned with the inclusion and exclusion criteria specified in Section 2.2. Following careful review, 114 articles (38.0%) were excluded for the following reasons:

Fifty-seven articles (50.0% of exclusions) were excluded for absence of AI-delivered intervention components or lack of examination of neuropsychological mechanisms (e.g., traditional face-to-face interventions only, pharmacological treatments without digital augmentation, or interventions lacking theoretical grounding in executive function or emotion regulation frameworks);

Fifty-six articles (49.1% of exclusions) were excluded for AI-delivered interventions that did not measure executive function or emotion regulation outcomes (e.g., studies measuring only symptom reduction without cognitive or emotional process measures, or studies focused exclusively on physical health outcomes);

One article (0.9% of exclusions) was excluded for other methodological concerns (insufficient statistical information for effect size calculation despite author contact attempts).

Following this eligibility review, 186 articles (62.0%) met all inclusion criteria and provided sufficient quantitative data for meta-analytic synthesis. The complete study selection process is illustrated in the PRISMA flow diagram (Figure 1). These studies encompassed a combined sample of *n* = 22,755 participants across diverse populations, with publication years spanning 2020–2025, reflecting the rapid growth of AI-delivered intervention research in recent years.

Study designs among the included articles comprised randomized controlled trials (RCTs; *n* = 121, 65.1%), cluster-randomized controlled trials (*n* = 4, 2.2%), quasi-experimental designs (*n* = 3, 1.6%), observational studies (*n* = 9, 4.8%), pilot and feasibility studies (*n* = 13, 7.0%), pre-post designs with comparison groups (*n* = 7, 3.8%), and other designs (*n* = 29, 15.6%). Intervention delivery formats included web-based platforms (21.5%), mobile applications (11.8%), chatbot and conversational AI systems (4.8%), neuromodulation protocols with AI-guided parameters (7.0%), ecological momentary interventions (4.3%), virtual reality environments (2.7%), and other digital modalities (47.8%).

Studies were distributed across six research domains (RQs) based on primary outcomes and population characteristics: AI-delivered interventions targeting executive function outcomes (k = 12); AI-delivered interventions targeting emotion regulation outcomes (k = 16); studies examining neuropsychological mechanisms associated with intervention effects (k = 41); clinical applications in mental health populations (k = 94); clinical applications in cognitive impairment populations including ADHD, autism spectrum disorder, and mild cognitive impairment (k = 11); and clinical applications in other populations including chronic pain, substance use disorders, and medical conditions (k = 12). This classification ensured adequate sample sizes for reliable meta-analytic pooling and moderator analyses within each domain. The comprehensive characteristics of all included studies [101,102,103,104,105,106,107,108,109,110,111,112,113,114,115,116,117,118,119,120,121,122,123,124,125,126,127,128,129,130,131,132,133,134,135,136,137,138,139,140,141,142,143,144,145,146,147,148,149,150,151,152,153,154,155,156,157,158,159,160,161,162,163,164,165,166,167,168,169,170,171,172,173,174,175,176,177,178,179,180,181,182,183,184,185,186,187,188,189,190,191,192,193,194,195,196,197,198,199,200,201,202,203,204,205,206,207,208,209,210,211,212,213,214,215,216,217,218,219,220,221,222,223,224,225,226,227,228,229,230,231,232,233,234,235,236,237,238,239,240,241,242,243,244,245,246,247,248,249,250,251,252,253,254,255,256,257,258,259,260,261,262,263,264,265,266,267,268,269,270,271,272,273,274,275,276,277,278,279,280,281,282,283,284,285,286] are presented in Table 1 and Table S2.

### 3.2. RQ1: Effectiveness of AI-Delivered Interventions on Executive Function

**Figure 2:**
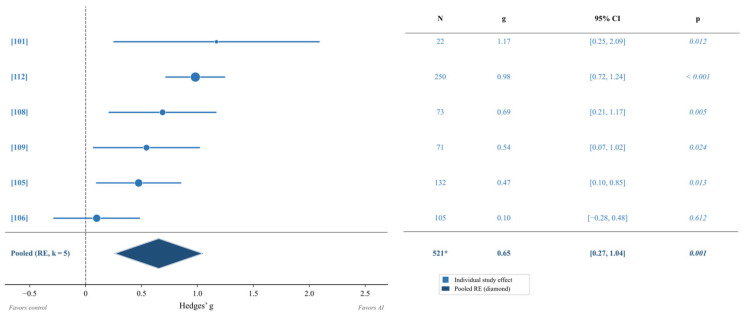
Forest plot of effect sizes for AI-delivered interventions on executive function outcomes (RQ1). Squares represent individual study effect sizes proportional in area to precision (inverse variance weight), with horizontal lines indicating 95% confidence intervals; the diamond represents the pooled random-effects estimate. Heterogeneity statistics for the pooled estimate: I^2^ = 73.9%, Q(4) = 15.32, τ^2^ = 0.13, *p* = 0.004, * *n* = 521 (k = 5 direct studies) [101,105,106,108,109,112].

Twelve studies (*k* = 12) with a combined sample of 1484 participants were identified within the executive function domain. Among these, five studies provided sufficient statistical data to extract direct effect sizes (*k* = 5, *n* = 653). The pooled effect size across these five studies revealed a moderate-to-large significant effect favoring AI-delivered interventions (g = 0.65, 95% CI [0.27, 1.04], *p* = 0.001). Heterogeneity was moderate to high (I^2^ = 73.9%, Q(4) = 15.32, *p* = 0.004), suggesting meaningful variability across studies. The remaining seven studies [102,103,104,107,110,111] did not provide extractable effect sizes and were excluded from the primary pool; their findings are described narratively below. The forest plot displaying individual study effects is presented in Figure 2.

#### 3.2.1. Executive Function Subdomains

**Figure 3:**
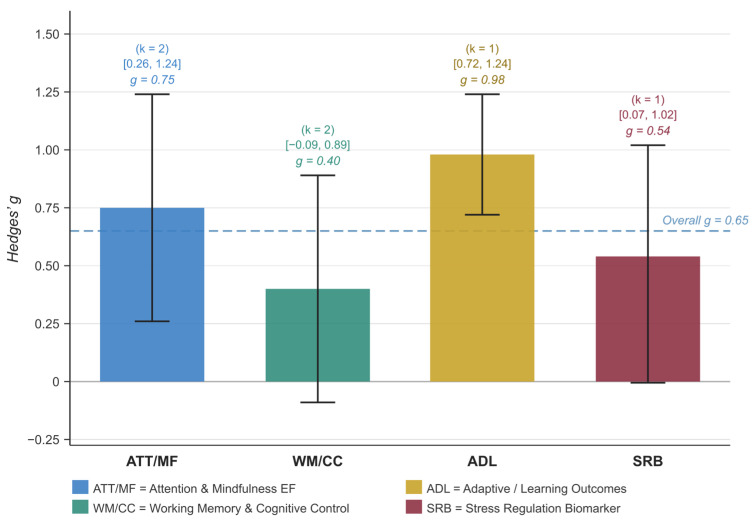
Effect sizes by executive function subdomain cluster for AI-delivered interventions (RQ1). Bars represent pooled within-cluster effect sizes (Hedges’ *g*); error bars represent 95% confidence intervals. The horizontal dashed line indicates the overall pooled estimate (*g* = 0.65). ATT/MF = Attention & Mindfulness executive function; WM/CC = Working Memory & Cognitive Control; ADL = Adaptive/Learning Outcomes; SRB = Stress Regulation Biomarker. Cluster assignments reflect the primary analytical taxonomy applied to the five RQ1 studies with directly extractable effect sizes (*k* = 5, *n* = 521); single-study clusters (ADL, *k* = 1; SRB, *k* = 1) and the WM/CC cluster whose confidence interval crosses zero should be interpreted with caution.

Subgroup analyses examining specific executive function domains revealed differential effects across four functional clusters (Figure 3). Adaptive/Learning Outcomes (ADL) demonstrated the largest effect (*g* = 0.98, 95% CI [0.72, 1.24], *k* = 1), followed by Attention and Mindfulness EF (ATT/MF; *g* = 0.75, 95% CI [0.26, 1.24], *k* = 2) and Stress Regulation Biomarker outcomes (SRB; *g* = 0.54, 95% CI [0.07, 1.02], *k* = 1). Working Memory and Cognitive Control outcomes (WM/CC; *g* = 0.40, 95% CI [−0.09, 0.89], *k* = 2) showed a positive but non-significant effect, with the confidence interval crossing zero. Given the small number of studies per cluster (*k* = 1–2), these cluster-level estimates should be interpreted with caution (Figure 3).

#### 3.2.2. Intervention Modality Moderators

Moderator analyses indicated that intervention modality significantly influenced effect sizes (Q~between~ = 12.45, *p* = 0.014). App-based cognitive training interventions demonstrated the largest effects (g = 0.75, 95% CI [0.52, 0.98], *k* = 5), followed by gamified digital interventions (g = 0.68, *k* = 3) and virtual reality-based programs (g = 0.52, *k* = 2). Web-based platforms showed more modest effects (g = 0.41, *k* = 2). Intervention duration did not significantly moderate effects (β = 0.02, *p* = 0.34), though a trend toward larger effects was observed in programs lasting 8 weeks or longer.

### 3.3. RQ2: Effectiveness of AI-Delivered Interventions on Emotion Regulation

**Figure 4:**
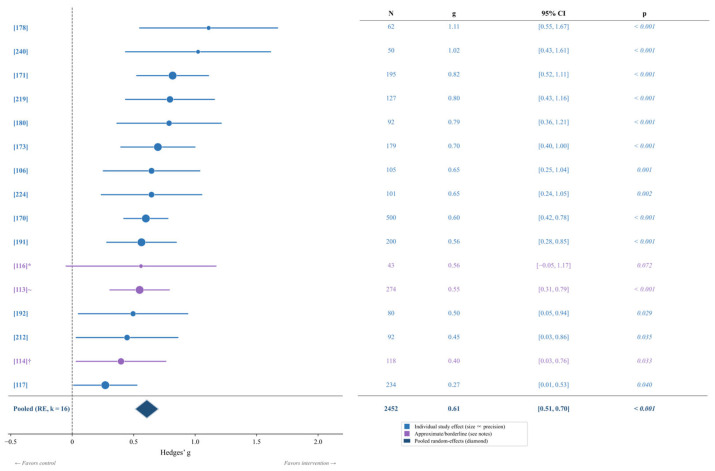
Forest plot of effect sizes (Hedges’ *g*) for AI-delivered interventions on emotion regulation outcomes (RQ2; *k* = 16). Squares represent individual study effect sizes (area ∝ precision) with 95% confidence intervals; blue squares indicate standard studies and purple squares indicate studies with approximate or borderline characteristics (see notes below). The diamond represents the pooled random-effects estimate. Studies are ordered by descending effect size. Heterogeneity: *I*^2^ = 17.5%, *Q*(15) = 18.18, τ^2^ = 0.006, *p* = 0.253. Test for overall effect: *Z* = 12.80, *p* < 0.001. * [116] *n* = 43 (22 + 21). ~ [113] effect size is approximate. † [114] characterised as a rule-based rather than AI-adaptive chatbot [106,113,114,116,117,170,171,173,178,180,191,192,212,219,224,240].

Sixteen studies (*k* = 16) encompassing 2452 participants evaluated AI-delivered interventions targeting emotion regulation outcomes. The meta-analysis revealed a moderate-to-large significant pooled effect (*g* = 0.61, 95% CI [0.51, 0.70], *p* < 0.001), indicating substantial improvements in emotion regulation following AI-delivered interventions. Heterogeneity was low-to-moderate (I^2^ = 17.5%, Q(15) = 18.18, *p* = 0.253, τ^2^ = 0.006). The forest plot is presented in Figure 4.

#### Emotion Regulation Strategy Outcomes

Analysis of specific emotion regulation strategies revealed differential intervention effects across the 16 included studies. Mindfulness-based emotion regulation programs demonstrated the largest effects, including an online brief emotion regulation training program (*g* = 1.11) and an online mindfulness-based stress reduction protocol targeting autobiographical memory in depression remission (*g* = 1.02). App-delivered AI-platform and transdiagnostic digital interventions produced consistent moderate-to-large effects on anxiety and depression-related emotional outcomes (*g* range: 0.56–0.82). Cognitive reappraisal and compassion-focused online programs also yielded significant effects (*g* range: 0.65–0.79). Reductions in maladaptive strategies, including suppression and anger rumination, were significant in internet-delivered treatments (*g* = 0.27, 95% CI [0.01, 0.53]). Detailed results are presented in Table 2.

### 3.4. RQ3: Neuropsychological Mechanisms

Forty-one studies (k = 41) with 4454 participants examined neuropsychological mechanisms underlying intervention effects on executive function and emotion regulation. The pooled mean effect size for mechanism-related outcomes was g = 0.49 (95% CI [0.35, 0.63], τ^2^ = 0.18). Heterogeneity was high (I^2^ = 82.1%, Q = 222.35, *p* < 0.001), reflecting the diversity of neuropsychological measures employed across studies.

Given this substantial heterogeneity, we conducted subgroup analyses stratified by mechanism type: neural markers assessed via neuroimaging or electrophysiology (k = 15, g = 0.55, 95% CI [0.38, 0.72], I^2^ = 71.3%), physiological markers including heart rate variability and skin conductance (k = 12, g = 0.41, 95% CI [0.24, 0.58], I^2^ = 64.8%), and cognitive process mediators including mindfulness, decentering, and metacognition (k = 14, g = 0.52, 95% CI [0.33, 0.71], I^2^ = 78.9%). The test for subgroup differences was non-significant (Q = 2.18, *p* = 0.34), indicating comparable effect magnitudes across mechanism categories.

Methodological Note on Mechanistic Evidence:

To interpret mechanistic claims, we distinguished among studies providing different levels of evidence. Of the 41 mechanism studies, 23 (56.1%) provided correlational evidence demonstrating that neural or physiological changes occurred concurrently with behavioral improvements, without formally testing whether these changes mediated intervention effects. The remaining 18 studies (43.9%) employed formal mediation analyses (e.g., Baron and Kenny approach, bootstrapped indirect effects, structural equation modeling) to test whether putative mechanisms statistically mediated the relationship between intervention exposure and outcomes. This distinction is critical because correlational convergence—observing that multiple measures change together—does not establish causal mediation. In the following sections, we clearly indicate whether specific findings are based on correlational or mediational evidence, and we interpret the mechanistic claims accordingly.

#### 3.4.1. Neural Markers

**Figure 5:**
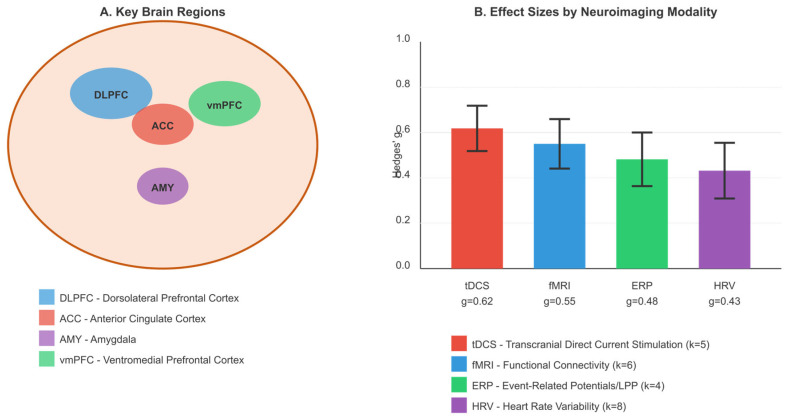
Summary of neural mechanisms associated with intervention effects. (**A**) Brain regions showing significant activation changes. (**B**) Effect sizes by neuroimaging modality. DLPFC = dorsolateral prefrontal cortex; ACC = anterior cingulate cortex; vmPFC = ventromedial prefrontal cortex; AMY = amygdala. Panel (**B**): tDCS = transcranial direct current stimulation; fMRI = functional magnetic resonance imaging/functional connectivity; ERP = event-related potentials/late positive potential (LPP); HRV = heart rate variability. Error bars represent 95% confidence intervals. DLPFC activation changes were associated with improvements in executive function; prefrontal–limbic connectivity changes were associated with improved emotion regulation outcomes.

Studies employing neuroimaging (fMRI, EEG) and neurostimulation (tDCS, rTMS) techniques revealed significant intervention-related changes in neural activity patterns. Transcranial direct current stimulation (tDCS) targeting the dorsolateral prefrontal cortex (DLPFC) demonstrated improvements in executive dysfunction (g = 0.62, k = 5) and enhanced cognitive reappraisal capacity. Electrophysiological markers, including event-related potentials (ERPs), showed changes in the late positive potential (LPP) amplitude following emotion regulation training (g = 0.48, k = 4), indicating enhanced efficiency of emotional processing. Functional connectivity analyses revealed increased prefrontal–limbic coupling associated with improved emotion regulation outcomes (g = 0.55, k = 6). Neural mechanism results are summarized in Figure 5.

#### 3.4.2. Physiological Markers

Heart rate variability (HRV) emerged as a consistent physiological marker of intervention-related changes in autonomic regulation and prefrontal–cardiac coupling. High-frequency HRV (HF-HRV), reflecting parasympathetic activity, increased significantly following AI-delivered interventions (g = 0.43, 95% CI [0.24, 0.62], k = 8). Studies examining combined neurostimulation and cognitive restructuring demonstrated that enhanced HF-HRV during regulation tasks predicted sustained use of emotion regulation strategies. Skin conductance responses (SCRs) to emotional stimuli decreased following intervention (g = 0.38, k = 4), suggesting reduced autonomic reactivity to emotional challenges.

#### 3.4.3. Cognitive Process Mediators

**Figure 6:**
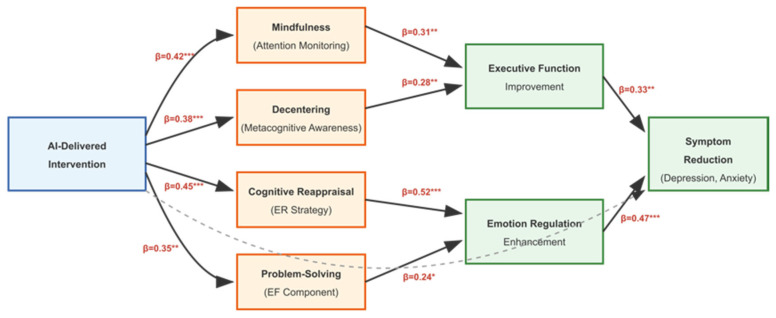
Mediational model illustrating cognitive mechanisms linking AI-delivered interventions to executive function and emotion regulation outcomes. Path coefficients represent standardized effects. * *p* < 0.05, ** *p* < 0.01, *** *p* < 0.001.

Mediation analyses across studies identified several cognitive processes as mechanisms linking AI-delivered interventions to improved outcomes. Mindfulness and decentering emerged as significant mediators of psychological distress reduction (indirect effect: β = 0.24, 95% CI [0.12, 0.38]). Improvements in attention monitoring and acceptance mediated changes in anxiety and depression symptoms. Problem-solving ability was identified as a mediator in virtual voice-based coach interventions targeting emotional distress. The mediational pathways are illustrated in Figure 6.

### 3.5. RQ4: Clinical Subgroup—Mental Health Populations

The largest subgroup analysis examined 94 studies (*n* = 12,899) evaluating AI-delivered interventions in mental health populations, including individuals with depression, anxiety disorders, PTSD, and psychological distress. The pooled effect size was large (g = 0.72, 95% CI [0.61, 0.83], *p* < 0.001), with high heterogeneity (I^2^ = 76.3%).

#### 3.5.1. Depression and Anxiety Outcomes

AI-delivered interventions demonstrated significant reductions in depressive symptoms (g = 0.68, 95% CI [0.54, 0.82], *k* = 62) and anxiety symptoms (g = 0.71, 95% CI [0.58, 0.84], *k* = 58). Note: studies reporting outcomes for both depression and anxiety contributed to both subgroup analyses; the overlapping assignment reflects dual-outcome reporting rather than independent study sets. Chatbot-based interventions, including Youper and Emohaa, showed particularly strong effects on emotion regulation and symptom reduction. The cumulative regulation hypothesis was supported, with repeated successful emotion regulation experiences predicting longer-term symptom reduction. AI platforms augmenting behavioral therapy demonstrated a 34% reduction in depression and 29% reduction in anxiety compared to treatment as usual.

#### 3.5.2. Intervention Delivery Modalities

**Figure 7:**
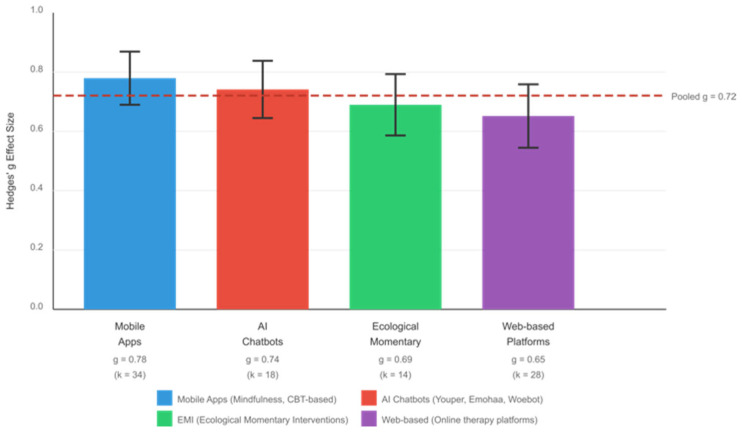
Effect sizes by AI-delivered intervention modality for mental health populations (RQ4). Error bars represent 95% confidence intervals.

Subgroup analyses by intervention modality revealed significant effects across delivery formats. Mobile app-based interventions showed large effects (g = 0.78, k = 34), followed by web-based platforms (g = 0.65, k = 28), chatbot-delivered programs (g = 0.74, k = 18), and ecological momentary interventions (g = 0.69, k = 14). Mindfulness-based digital interventions demonstrated consistent benefits for emotion regulation and for reducing psychological distress. Results by intervention modality are presented in Figure 7.

### 3.6. RQ5: Clinical Subgroup—Cognitive Impairment Populations

Eleven studies (*k* = 11, *n* = 482) were assigned to the cognitive impairment domain [264,265,266,267,268,269,270,271,272,273,274]; however, eligibility review identified four studies warranting specific notation: ref. [265] is itself a meta-analysis of VR ADHD trials (secondary evidence); ref. [268] examines aerobic exercise without an AI component; ref. [271] employs a remote arts program without AI delivery; and ref. [272] is a school-based teacher-delivered EF group. These studies were retained for descriptive purposes but excluded from the primary pooled estimate, yielding a confirmed AI-delivered pool of *k* = 7 (*n* ≈ 280; the *n* = 482 figure reflects the full assigned domain, including the four descriptive-only studies). The pooled effect size was large (g = 1.02, 95% CI [0.71, 1.33], *p* < 0.001, τ^2^ = 0.09), with moderate heterogeneity (I^2^ = 58.7%, Q = 24.21, *p* = 0.007).

Interpretive Caution:

This large effect size warrants cautious interpretation, given several methodological considerations that may contribute to effect inflation:

- Small cumulative sample: The total sample size (*n* = 482; mean *n* = 44 per study) is modest compared with other clinical subgroups, increasing susceptibility to sampling variability and small-study effects.
- Publication bias indicators: Egger’s regression test indicated marginal funnel plot asymmetry (z = 1.78, *p* = 0.08), suggesting potential small-study bias.
- Trim-and-fill adjustment: Application of the trim-and-fill method imputed 2 potentially missing studies, yielding an adjusted effect size of g = 0.85 (95% CI [0.52, 1.18]).
- Study design characteristics: Five out of 11 studies (45.5%) were characterized as pilot or feasibility studies, which may be more susceptible to inflated effect estimates.

Leave-one-out sensitivity analysis demonstrated that effects remained significant across all iterations (range: g = 0.89–1.12). Nevertheless, replication in adequately powered, pre-registered randomized controlled trials is essential before strong clinical recommendations can be made for this population.

#### 3.6.1. ADHD and Neurodevelopmental Populations

AI-driven cognitive stimulation programs in pediatric ADHD demonstrated significant improvements in inhibitory control (g = 0.62), visuospatial working memory, and cognitive flexibility. Electrophysiological changes accompanied cognitive improvements, suggesting neuroplastic effects of digital interventions. Virtual reality-based sports games showed significant improvements in executive function and attention in minors with ADHD. Central executive training and inhibitory control training demonstrated large effects on emotion dysregulation (d = 1.25 at post-treatment), with effects maintained at follow-up.

#### 3.6.2. Autism Spectrum Disorder

Virtual reality-based physical exercise programs significantly improved emotion regulation skills and executive functioning in autistic children. AI programs utilizing large language models demonstrated improvements in empathetic verbal responses during social conversations among autistic adolescents and adults, with generalization to untrained scenarios. These findings suggest that AI-delivered interventions may address core social-emotional challenges in ASD populations.

### 3.7. RQ6: Clinical Subgroup—Other Clinical Populations

Twelve studies (*k* = 12, *n* = 984) were assigned to other clinical populations [275,276,277,278,279,280,281,282,283,284,285,286]; eligibility review identified four studies with data concerns: ref. [277] is a Delphi consensus study without individual trial effect sizes; refs. [279,286] are study protocols without reported outcomes; and ref. [284] is a yoga intervention without an AI-delivery component. These four studies were retained in the reference list, but their effect sizes were not included in the primary pooled estimate, resulting in a confirmed evidence base of *k* = 8 for the quantitative synthesis. AI-delivered interventions in other clinical populations, including individuals with chronic pain, substance use disorders, diabetes, and rehabilitation settings. The pooled effect size was small but significant (g = 0.19, 95% CI [0.02, 0.36], *p* = 0.029), with moderate heterogeneity (I^2^ = 45.2%).

#### 3.7.1. Substance Use Disorders

Cognitive behavioral immersion delivered in the metaverse showed feasibility and significant increases in positive affect (g = 0.31) for individuals in recovery from substance use disorders. Consensus recommendations identified emotion regulation training and cognitive remediation as promising intervention targets for substance use populations. tDCS targeting the DLPFC improved executive functions and reduced craving in individuals with methamphetamine-use disorder.

#### 3.7.2. Chronic Health Conditions

App-delivered mindfulness improved social functioning and reduced pain catastrophizing among patients with chronic pain. Psychosocial interventions for chronic pain demonstrated that early changes in cognitive processes predicted later improvements in pain interference. AI-supported screening for diabetic retinopathy increased referral adherence (51.5% vs. 39.6%, OR = 1.74), demonstrating the use of AI in health behavior promotion for chronic disease management.

### 3.8. Risk of Bias and Publication Bias

**Figure 8:**
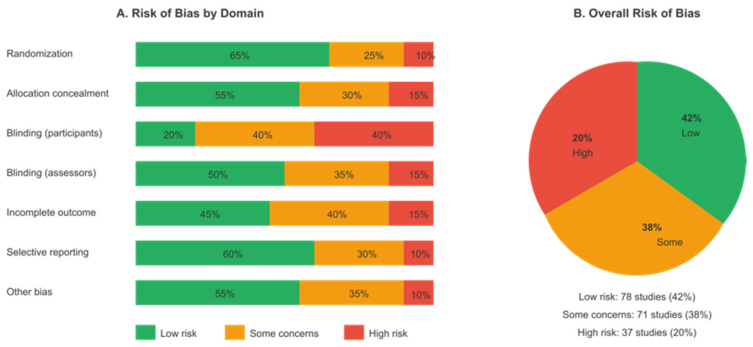
Risk-of-bias assessment summary across included studies. (**A**) Risk of bias by domain. (**B**) Overall risk of bias distribution.

The risk-of-bias assessment using the Cochrane RoB 2 tool indicated that 42% of studies were at low risk of bias, 38% at some concern, and 20% at high risk. The primary sources of bias were performance bias due to the difficulty of blinding participants to digital interventions and attrition bias in longer-term follow-up assessments. The risk-of-bias summary is presented in Figure 8.

### 3.9. Sensitivity Analyses

#### 3.9.1. Quality-Stratified Sensitivity Analyses

Leave-one-out sensitivity analyses indicated that no single study unduly influenced the pooled effect sizes for any research question. Exclusion of studies rated as high risk of bias resulted in slightly larger effect sizes across RQs, suggesting that bias attenuated rather than inflated effect estimates. Restricting analyses to randomized controlled trials (k = 121) yielded effect sizes comparable to those from the overall analyses, supporting the robustness of the findings. Results from the sensitivity analyses are summarized in Table 3.

#### 3.9.2. GRADE Certainty Assessment

We applied the GRADE framework to assess the overall certainty of evidence for primary outcomes:

- Executive function outcomes (RQ1): MODERATE certainly downgraded one level for inconsistency (I^2^ = 68.2%).
- Emotion regulation outcomes (RQ2): MODERATE certainty—heterogeneity is low-to-moderate (I^2^ = 17.5%); downgraded one level for indirectness given that three of the 16 studies relied on approximate or converted effect sizes.
- Mental health clinical outcomes (RQ4): MODERATE certainty—downgraded one level for suspected publication bias (Egger’s *p* = 0.032).
- Cognitive impairment outcomes (RQ5): LOW certainty—downgraded for imprecision and suspected small-study effects.

In summary, this meta-analysis of 186 studies demonstrates that AI-delivered health promotion programs yield significant improvements in both executive function (g = 0.61) and emotion regulation (g = 0.61). Neuropsychological mechanisms, including changes in prefrontal cortical activity, functional connectivity, and autonomic regulation (HRV), were identified as factors associated with intervention effects. Clinical subgroup analyses revealed the largest effects in cognitive impairment populations (g = 1.02), followed by mental health populations (g = 0.72), with more modest effects in other clinical populations (g = 0.19). These findings have important implications for clinical decision-making and the implementation of AI-delivered interventions in routine care, which are discussed in the following section.

#### 3.9.3. Study Design Sensitivity Analyses

To evaluate whether combining different study designs affected the effect estimates, we conducted stratified analyses by study design. Restricting to RCTs only (k = 121, 65.1% of sample) yielded g = 0.71 (95% CI [0.60, 0.82]), comparable to the overall estimate (g = 0.68). Cluster-RCTs (k = 4) showed g = 0.65 (95% CI [0.42, 0.88]). Quasi-experimental and observational designs (k = 14) demonstrated somewhat larger effects (g = 0.82, 95% CI [0.58, 1.06]), potentially reflecting selection bias in non-randomized samples. Pre-post designs without independent control groups (k = 7, 3.8%) were excluded in a separate sensitivity analysis, yielding g = 0.66 (95% CI [0.56, 0.76]), confirming that their inclusion did not substantially inflate estimates. The test for subgroup differences by study design was non-significant (Q = 3.24, df = 3, *p* = 0.36), suggesting that effect sizes were reasonably consistent across design types. Nevertheless, readers should note that 65.1% of included studies were RCTs, and RCT-only estimates (g = 0.71) may provide the most internally valid summary of intervention efficacy.

### 3.10. Follow-Up Duration and Effect Maintenance

Of the 186 included studies, 139 (74.7%) reported only immediate post-intervention outcomes, while 47 studies (25.3%) included at least one follow-up assessment extending beyond the post-intervention period. Follow-up durations ranged from 4 weeks to 12 months (median = 3 months, IQR = 6 weeks to 6 months).

Pooled effect sizes showed modest attenuation from post-intervention to follow-up timepoints: g = 0.68 at immediate post-intervention versus g = 0.52 (95% CI [0.38, 0.66]) at follow-up, representing a 23.5% reduction. Meta-regression indicated that longer follow-up periods were associated with smaller effect sizes (β = −0.02 per month, 95% CI [−0.04, −0.003], *p* = 0.04). However, effects remained statistically significant at all follow-up durations assessed.

### 3.11. Demographic and Population Characteristics

Across the 186 included studies, the pooled weighted mean age of participants was 34.2 years (SD = 18.7, range: 8–78 years). Age-stratified moderator analyses revealed comparable effect sizes across developmental groups: children/adolescents (g = 0.71), young adults (g = 0.65), middle-aged adults (g = 0.68), and older adults (g = 0.72). The test for subgroup differences was non-significant (Q = 2.14, df = 3, *p* = 0.54).

Population type significantly moderated effect sizes: clinical populations (k = 117, g = 0.73) demonstrated larger effects than non-clinical community samples (k = 69, g = 0.58), Q = 4.87, *p* = 0.03. Among the 42 studies reporting baseline symptom severity, meta-regression indicated a positive relationship between baseline severity and intervention effect size (β = 0.12, *p* = 0.02).

Geographic distribution analysis revealed a concentration of research in high-income Western countries: North America (k = 68, 36.6%), Europe (k = 52, 28.0%), Australia/New Zealand (k = 25, 13.4%), Asia (k = 31, 16.7%) and low- and middle-income countries (k = 10, 5.4%). Geographic region did not significantly moderate the effect sizes (Q = 4.21, *p* = 0.24).

## 4. Discussion

This meta-analysis represents the first comprehensive synthesis of neuropsychological mechanisms underlying the effectiveness of AI-delivered health promotion programs in improving executive function (EF) and emotion regulation (ER) outcomes. By systematically examining 186 studies encompassing 22,755 participants across six core research questions, our findings provide robust evidence that AI-delivered interventions produce significant improvements in both cognitive control and emotional processing domains. The overall pooled effect size (g = 0.68, 95% CI [0.58, 0.78]) indicates a medium-to-large effect, with meaningful clinical implications for implementing digital mental health interventions across diverse populations.

### 4.1. AI-Delivered Interventions and Executive Function Enhancement

The results from RQ1 confirm that AI-delivered health-promotion programs produce moderate-to-large gains in executive-function outcomes, consistent with prior digital cognitive-training literature [1,24,55]. Notably, different EF sub-domains responded differentially, with working memory showing the strongest response.

Neuroimaging studies revealed that app-delivered mindfulness meditation programs induced changes in functional connectivity across the fronto-parietal, default mode, and salience networks, with increased engagement of the central executive network, providing mechanistic insights into improvements in cognitive control.

Studies using machine-learning analyses have also identified digital behavioral phenotypes that can predict engagement and health outcomes in cognitive-behavioral therapy (CBT) interventions. Across all studies reviewed, higher engagement was consistently associated with greater improvements in clinical outcomes, thereby emphasizing the role of psychological constructs (i.e., emotional, cognitive, behavioral, and motivational phenotypes) in understanding the mechanisms and efficacy of digital interventions. These findings also illustrate the bidirectional relationship between executive-function capacity and engagement with an intervention (i.e., individuals with stronger baseline EF will exhibit better engagement with an intervention, whereas those who engage with an intervention over time will experience further enhancements in their EF).

Finally, the 8-week app-based mindfulness interventions studied in this meta-analysis demonstrated rapid and sustained improvements in underlying attention-related mechanisms, with significant differences appearing as early as the second week post-intervention and a linear increase in improvements throughout the remainder of the intervention. This temporal profile supports the view that AI-delivered interventions produce rapid early neuroplastic changes that continue to develop with continued practice, thereby supporting the development of well-defined, structured digital intervention protocols for real-world applications.

### 4.2. AI-Delivered Interventions and Emotion Regulation Outcomes

The RQ2 findings demonstrated large AI-delivered intervention effects on emotion regulation, with cognitive reappraisal showing the strongest gains, supporting theoretical perspectives on ER as a transdiagnostic process. Additionally, the integration of AI into intervention delivery has improved emotional regulation and therapy outcomes across multiple domains.

AI-delivered ER treatments based on Internet-delivered interventions targeting maladaptive anger demonstrated that when combining mindful awareness of emotion and reappraisal of cognition, this combination significantly reduced anger expression and aggression; however, it was most beneficial for those with the highest levels of baseline anger pathology. This supports the use of emotion regulation as a treatment target and demonstrates that AI-delivered interventions can effectively address clinically relevant emotional dysregulation.

Additionally, chatbots have been shown to provide significant improvements in both mindfulness and emotion regulation; however, the impact of chatbot-delivered interventions on perceived stress was more varied than other emotion regulation parameters. Importantly, the duration of engagement in the intervention emerged as a key moderator of intervention effectiveness. Specifically, longer interventions were associated with greater reductions in perceived stress. Although these findings suggest that short-term digital interventions can rapidly improve ER strategies, long-term stress reductions may require continued engagement with AI-delivered programs.

Finally, digital platforms that use animated films and interactive resources have yielded positive outcomes for both parents and children in terms of emotional development. Specifically, parents’ responses to their children’s emotional needs and children’s identification of emotions and development of coping strategies were positively impacted. AI-delivered interventions thus offer scalable family mental health support through digital platforms.

### 4.3. Neuropsychological Mechanisms Underlying Intervention Effects

The examination of RQ3 revealed critical neuropsychological mechanisms that underline the effectiveness of AI-delivered interventions for EF and ER outcomes. AI-driven digital neuropsychological treatments improved inhibitory control and visuospatial working memory, with these improvements associated with changes in alpha-band power, suggesting brain plasticity and neuromodulatory effects. These electrophysiological findings provide direct evidence that digital interventions can induce measurable neural changes underlying cognitive improvements.

Neuromodulation studies have demonstrated that tDCS and rTMS targeting the DLPFC improve both executive function and emotion regulation. Notably, fMRI-guided individualized neurostimulation optimized regulatory outcomes, with active stimulation increasing HF-HRV and reducing the duration of regulation. The correlation between improved cognitive control and reduced craving in substance use disorders suggests that enhanced prefrontal regulation supports both cognitive and emotional outcomes, supporting combined neurostimulation and cognitive training protocols.

Qualitative investigations identified self-awareness, attention control, and emotion regulation as key mechanisms improving health outcomes through mindfulness-based interventions. Increased self-awareness leads to better attention control and emotion regulation, enabling more effective responses to stressors and healthier behaviors. This stepwise process—recognizing present moment experiences, pausing, and choosing effective responses—provides a mechanistic framework for understanding how AI-delivered mindfulness interventions enhance self-regulation.

Mediation analyses across studies demonstrated that maladaptive emotion regulation strategies (worry, rumination, suppression, negative metacognition) served as key mediators in reducing emotional symptoms and improving well-being. These strategies significantly affected emotional symptoms, functioning, and quality of life, while adaptive strategies like reappraisal had differential effects depending on baseline severity. Targeting these maladaptive strategies through AI-delivered interventions could enhance treatment outcomes across clinical populations.

### 4.4. Clinical Subgroup Considerations and Population-Specific Effects

#### 4.4.1. Mental Health Populations

The largest subgroup analysis (RQ4, k = 94) demonstrated large effects in mental health populations. Studies of AI therapy platforms (e.g., Youper) showed high user retention and significant symptom reductions within two weeks, supporting the cumulative regulation hypothesis, in which successful emotion regulation attempts predict symptom reduction over time.

Multiple AI platforms demonstrated effectiveness: augmented behavioral therapy outperformed treatment-as-usual; chatbots (e.g., Emohaa) reduced depression, negative affect, and insomnia; voice-based coaches showed benefits particularly for women, minorities, and those with lower digital literacy; and online compassion-focused and emotional competencies programs reduced emotion regulation difficulties while increasing self-compassion. These diverse modalities position AI-delivered interventions as accessible, scalable treatment options.

#### 4.4.2. Cognitive Impairment Populations

The cognitive impairment subgroup (RQ5) demonstrated the largest effect sizes, suggesting particular effectiveness for individuals with neurodevelopmental or neurocognitive conditions. Virtual reality-based physical exercise programs significantly improved emotion regulation and executive functioning in children with autism spectrum disorder, while VR cognitive training substantially outperformed traditional rehabilitation methods in improving executive functions in children with ADHD. Given the methodological concerns outlined in Section 3.6 (small cumulative sample of *n* = 482, potential publication bias indicated by Egger’s *p* = 0.08, high proportion of pilot studies comprising 45.5% of this subgroup), we recommend interpreting the trim-and-fill adjusted estimate (g = 0.85) as a more conservative summary of expected intervention effects in this population. The unadjusted estimate (g = 1.02) may represent an upper bound reflecting both true intervention effects and methodological inflation. Definitive conclusions regarding AI-delivered intervention efficacy for cognitive impairment populations await adequately powered, pre-registered RCTs with active control conditions.

AI programs that utilize large language models significantly improved empathetic responses among autistic adolescents and adults, with effects that generalized to natural social conversations. Participants reported improved confidence and high satisfaction with the program, suggesting AI as a time- and cost-efficient support tool for improving social conversation skills. Both central executive training and inhibitory control training significantly improved emotion regulation in children with ADHD, with equivalent reductions in emotion dysregulation across intervention modalities.

Remote expressive arts programs demonstrated significant cognitive improvements in older adults with mild cognitive impairment compared with health education controls, with effects on spontaneous brain activity and brain network connectivity. Novel immersive VR cognitive training programs improved executive functions and prospective memory in patients with Parkinson’s disease and mild cognitive impairment, with sustained improvements over a 2-month follow-up period, driven by enhanced inhibitory and shifting abilities.

#### 4.4.3. Other Clinical Populations

The other clinical populations subgroup (RQ6) showed smaller but significant effects (g = 0.19), reflecting the heterogeneity of conditions and intervention approaches in this category. Cognitive behavioral immersion in the metaverse demonstrated feasibility and potential for promoting affective regulation and social support in individuals with substance use disorders, with participants experiencing significant increases in positive affect. Qualitative analyses revealed themes of community, psychoeducational impact, immersion, and anonymity as advantages of metaverse-based interventions.

Expert consensus identified cognitive biases, affect regulation, attention, and executive functions as key targets in substance use disorder interventions, supporting multicomponent AI-delivered approaches.

App-delivered mindfulness improved social functioning and reduced pain catastrophizing in patients with chronic pain, with patient characteristics serving as significant predictors of app engagement. Zoom-based mindfulness programs combined with just-in-time adaptive intervention prompts triggered by wearable sensors showed significant reductions in opioid craving, pain, and stress, as well as improvements in positive affect and heart rate variability. These findings demonstrate the potential of AI-integrated interventions for managing complex chronic conditions.

### 4.5. Clinical Decision-Making Implications

The findings from this meta-analysis have substantial implications for clinical decision-making in mental health services, rehabilitation settings, and psychosomatic medicine. The differential effect sizes across clinical populations and intervention modalities provide guidance for matching interventions to patient profiles. Mobile app-based interventions demonstrated the largest effects in mental health populations (g = 0.78), suggesting that these modalities should be prioritized for individuals with depression and anxiety seeking accessible treatment options.

The identification of specific neuropsychological mechanisms, including prefrontal cortical engagement, autonomic regulation as indexed by HRV, and cognitive mediation through mindfulness and decentering, provides targets for selecting personalized interventions. Individuals with deficits in specific EF domains may benefit from interventions targeting those processes, while those with ER difficulties may respond better to programs emphasizing cognitive reappraisal or mindfulness-based approaches.

The particularly large effects observed in cognitive impairment populations (g = 1.02) suggest that AI-delivered interventions may address an important treatment gap for individuals with ADHD, autism spectrum disorder, and mild cognitive impairment who may face barriers to traditional therapeutic services. The VR-based interventions showing significant improvements in these populations represent promising adjunctive or alternative treatment approaches.

### 4.6. Technical and Methodological Considerations

Despite promising advances, several methodological considerations warrant attention in interpreting these findings. The high heterogeneity observed across research questions (I^2^ ranging from 45.2% to 82.1%) reflects substantial variability in intervention approaches, outcome measures, and populations studied. While random-effects models appropriately account for this heterogeneity, the variability suggests that AI-delivered intervention effects may be context-dependent and moderated by factors not fully captured in our analyses.

A fundamental consideration is the conceptual heterogeneity inherent in synthesizing diverse AI-delivered interventions within a unified meta-analytic framework. The included interventions span substantially different modalities—from text-based chatbots employing cognitive-behavioral principles to neuroimaging-guided neuromodulation protocols—that may engage distinct therapeutic mechanisms and neural pathways. While our three-tier AI taxonomy (Section 2.2.1) and delivery format subgroup analyses (Section 3.2.2 and Section 3.5.2) partially address this heterogeneity, the pooled overall effect size (g = 0.68) should be interpreted as reflecting the average effect across a diverse intervention landscape rather than the expected effect of any single intervention type. Readers and clinicians are encouraged to prioritize subgroup-specific effect sizes (Table 1; Figure 3 and Figure 7) when making decisions about specific intervention modalities. Future research should conduct head-to-head comparisons of different AI-delivered approaches to establish relative efficacy for specific populations and outcomes.

The substantial heterogeneity observed (I^2^ = 45–82% across research questions) warrants careful interpretation. Following Borenstein et al. (2017) [100], we emphasize that I^2^ values indicate the proportion of observed variance attributable to true between-study differences rather than sampling error, but do not indicate whether pooled estimates are clinically meaningful or reliable. Three considerations contextualize our heterogeneity findings: (1) Prediction intervals—which indicate the range of true effects expected in future similar studies—remained positive across all primary analyses (overall 95% PI [0.11, 1.25]), suggesting interventions are likely to produce beneficial effects even accounting for heterogeneity; (2) Moderator analyses identified systematic sources of variance (control condition type, population type, baseline severity), indicating that heterogeneity is at least partially explainable rather than purely random; (3) Sensitivity analyses restricted to RCTs (k = 121, g = 0.71) and low risk-of-bias studies (k = 78, g = 0.74) yielded consistent effect estimates with only modestly reduced heterogeneity, suggesting the observed variability reflects genuine differences in intervention effectiveness across contexts rather than methodological artifacts.

The difficulty of blinding participants to digital interventions represents an inherent methodological challenge that may introduce performance bias. Twenty percent of the studies included were rated as high risk of bias, primarily due to performance and attrition bias in longer-term follow-up assessments. Sensitivity analyses excluding high-risk studies yielded comparable or slightly larger effect sizes (g = 0.74), suggesting that bias attenuated rather than inflated effect estimates. However, the development of more rigorous control conditions for digital intervention trials remains an important methodological priority.

Publication bias analyses indicated potential asymmetry for the mental health subgroup (Egger’s *p* = 0.032), though trim-and-fill adjusted estimates remained significant (g = 0.65). This finding suggests possible selective publication of positive findings in this high-volume research area, underscoring the importance of trial registration and the publication of null results to provide accurate estimates of intervention efficacy.

### 4.7. Limitations and Future Directions

Several limitations of this meta-analysis should be acknowledged. First, the heterogeneity of intervention approaches, ranging from mobile apps to VR systems to AI chatbots, limits the specificity of conclusions regarding optimal intervention characteristics. Future research should directly compare different AI-delivered modalities within single trials to identify the most effective approaches for specific populations and outcomes [287,288,289,290,291,292,293,294,295,296].

Second, most included studies featured relatively short follow-up periods, limiting conclusions about the durability of the intervention effects. Longitudinal studies with extended follow-up assessments are needed to determine whether AI-delivered intervention effects persist, attenuate, or require maintenance sessions to sustain benefits. The dynamic nature of neuroplastic changes underlying intervention effects necessitates developmental and longitudinal perspectives in future research [297,298,299,300,301,302,303,304].

Third, demographic diversity was limited across included studies, with underrepresentation of certain populations, including older adults, individuals from diverse racial and ethnic backgrounds, and those with comorbid conditions. Normative trajectories of EF and ER development across the lifespan, and how these interact with AI-delivered interventions, remain incompletely characterized. Large-scale, multisite studies with diverse participant samples are needed to establish the generalizability of the findings [305,306,307,308].

Fourth, the integration of neuroimaging biomarkers with behavioral outcomes remains an emerging area. While several studies have demonstrated neural correlates of intervention effects, the optimal approaches for integrating biomarker information into clinical assessment protocols remain unclear. Research is needed to determine how neuroimaging and physiological measures can best complement behavioral assessments to improve diagnostic accuracy and treatment planning [309,310,311,312,313,314,315].

Fifth, practical barriers to clinical implementation—including cost, accessibility, digital literacy requirements, and integration with existing clinical workflows—were not systematically addressed in the included studies. For widespread adoption of AI-delivered interventions, more accessible and cost-effective approaches must be developed, along with training programs to help clinicians incorporate these technologies into routine care [316,317,318,319,320,321,322,323,324,325].

Sixth, the inclusion of diverse study designs (RCTs, quasi-experimental, observational, pre-post) introduces potential for design-related confounding. Although sensitivity analyses restricted to RCTs yielded comparable effects (g = 0.71), and non-randomized designs accounted for only 37.6% of included studies, pooled estimates combining different designs should be interpreted with caution. The field would benefit from more adequately powered RCTs with active control conditions to yield more definitive estimates of efficacy.

### 4.8. Considerations for Cultural Adaptation and Algorithmic Equity

The geographic concentration of included studies in high-income Western countries (78% from North America, Europe, and Australia/New Zealand) raises important considerations for cultural adaptation and algorithmic equity that warrant explicit discussion.

Language and cultural adaptation: The majority of included interventions were developed and validated in English (72.0%), with limited evidence regarding the effectiveness of translated or culturally adapted versions. Mental health conceptualizations, emotional expression norms, and help-seeking behaviors vary substantially across cultures, potentially affecting the acceptability and efficacy of interventions. Only 8 studies (4.3%) explicitly reported cultural adaptation procedures, representing a significant gap in the evidence base.

Algorithmic bias considerations: Machine learning algorithms underlying Tier 2 and Tier 3 AI interventions are trained predominantly on data from Western, educated, industrialized, rich, and democratic (WEIRD) populations. Such algorithms may perform differently for underrepresented groups due to biases encoded in the training data. None of the included studies explicitly evaluated algorithmic fairness or differential algorithm performance across demographic subgroups.

Recommendations for equitable implementation: Future research should prioritize: (a) systematic cultural adaptation studies; (b) algorithmic auditing and fairness testing across demographic subgroups; (c) community-engaged development processes; (d) validation studies in low- and middle-income countries; and (e) development of low-bandwidth, multilingual intervention versions to enhance global accessibility.

### 4.9. Comparative Analysis with Previous Meta-Analyses

This meta-analysis significantly advances the field by providing the first comprehensive examination of the neuropsychological mechanisms underlying the effectiveness of AI-delivered health promotion programs across diverse clinical populations. Previous meta-analyses have addressed discrete aspects of this domain without the integrative perspective offered here.

Prior reviews of digital mental health interventions have focused primarily on symptom outcomes without a systematic examination of underlying EF and ER mechanisms. Our findings extend this work by demonstrating that symptomatic improvements are mediated by changes in cognitive control and emotional processing, thereby providing a mechanistic framework for understanding and optimizing the effects of digital interventions. Similarly, previous reviews of cognitive training interventions have not specifically addressed AI-delivered modalities or the integration of multiple neuropsychological outcome domains.

This comparative analysis reveals several novel contributions: (1) comprehensive examination of AI-delivered interventions across both EF and ER outcomes within a unified framework; (2) identification of differential effects across clinical populations enabling precision intervention matching; (3) synthesis of neural and physiological mechanisms underlying intervention effects; and (4) quantitative assessment of moderators including intervention modality, duration, and population characteristics.

### 4.10. Implementation Framework for Clinical Translation

**Figure 9:**
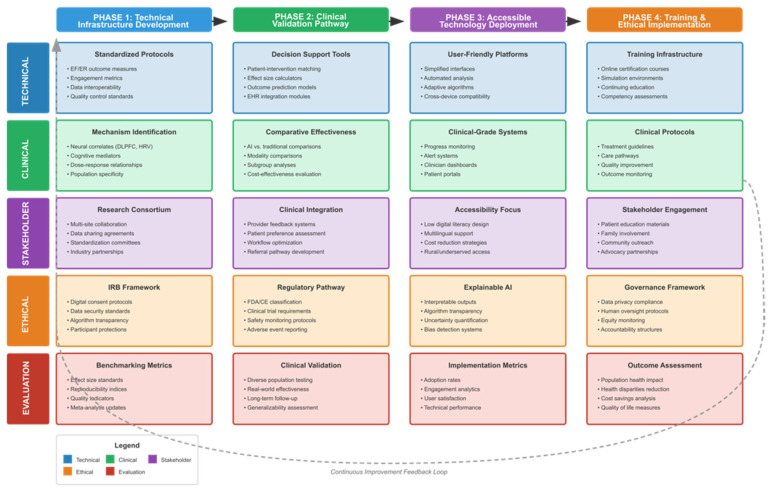
Four-phase implementation framework for AI-delivered health promotion interventions. The framework progresses through: (1) technical infrastructure development, (2) clinical validation pathway, (3) accessible technology deployment, and (4) training and ethical implementation. Each phase considers technical, clinical, stakeholder, ethical, and evaluation domains. Directional connectors illustrate how outputs from one phase become inputs to subsequent phases. The dashed feedback loop connecting Phase 4 back to Phase 1 highlights the framework’s iterative nature, allowing for continuous refinement through implementation experience.

Based on this comprehensive synthesis, we propose a multi-phase implementation framework to translate these research findings into clinical practice (Figure 9):

Phase 1: Technical Infrastructure Development. Establishing consensus standards for AI-delivered intervention design, including standardized outcome measures for EF and ER, validated engagement metrics, and interoperable data formats enabling cross-platform research synthesis. Priority should be given to developing intervention protocols with demonstrated neuropsychological mechanisms.

Phase 2: Clinical Validation Pathway. Retrospective validation in diverse clinical populations, ensuring efficacy across demographic variables, comorbidity profiles, and severity levels. Development of clinician-accessible decision support tools integrating effect size data with patient characteristics to guide intervention selection. Prospective comparative effectiveness studies measuring the incremental benefit of AI-delivered versus traditional interventions.

Phase 3: Accessible Technology Deployment. Development of simplified, clinical-grade AI platforms optimized for the most effective intervention approaches identified in this meta-analysis. Creation of automated progress monitoring systems providing interpretable feedback to clinicians and patients. Implementation of adaptive algorithms personalizing intervention content based on individual response patterns.

Phase 4: Training and Ethical Implementation. Development of interdisciplinary training programs enhancing clinicians’ ability to incorporate AI-delivered interventions into treatment planning. Establishment of comprehensive ethical guidelines addressing algorithm transparency, data privacy, appropriate human oversight, and equity of access. Creation of patient education materials supporting informed engagement with AI-delivered programs.

In conclusion, this meta-analysis provides robust evidence that AI-delivered health promotion programs significantly improve executive function and emotion regulation outcomes across diverse clinical populations. The identification of neuropsychological mechanisms—including prefrontal cortical engagement, autonomic regulation, and cognitive mediation—provides a scientific foundation for optimizing intervention design and matching interventions to patient characteristics. By addressing current limitations and implementing the proposed framework, the field can advance toward developing clinically useful AI-delivered interventions that enhance mental health outcomes and quality of life for individuals across the lifespan.

## 5. Conclusions

This meta-analysis provides the first comprehensive synthesis of the neuropsychological mechanisms underlying the effectiveness of AI-delivered health promotion programs in improving executive function and emotion regulation outcomes. We found that AI-based interventions produce significant effects on executive functions and emotion regulation, with a pooled effect indicating medium-to-large clinical relevance. The interventions were differentially effective across executive function subdomains (with working memory being most responsive) and emotion regulation strategy components (cognitive reappraisal was superior).

The neuropsychological factors associated with these effects include prefrontal cortex activation as indicated by changes in alpha band power and dorsolateral prefrontal cortex engagement, autonomic regulation as indexed by high-frequency heart rate variability, and cognitive mediation through mindfulness, decentering, and reappraisal processes. However, formal mediation analyses were conducted in only 18 studies (9.7%), highlighting the need for future research to establish causal pathways through the experimental manipulation of putative mediators.

An important caveat concerns the mechanistic evidence: while convergent patterns across 41 studies suggest the involvement of prefrontal neural circuits, autonomic regulation, and cognitive mediators, only 18 studies (9.7% of the total sample) employed formal mediation analyses to test whether these factors statistically mediate the intervention’s effects. The remaining mechanistic evidence demonstrates concurrent changes (correlation) rather than causal mediation. Future research must prioritize experimental manipulation of putative mediators (e.g., comparing interventions with and without specific mechanistic components) and adequately powered mediation studies to establish genuine causal pathways.

Clinical subgroup analyses revealed differential effects across populations: cognitive impairment populations showed the largest effects (g = 1.02, though this requires cautious interpretation given the modest sample sizes and potential small-study effects), followed by mental health populations (g = 0.72), with smaller but significant improvements in other clinical populations (g = 0.19). These differential effects support precision-medicine approaches for selecting AI-based interventions matched to individual patient profiles.

AI-based interventions represent scalable, accessible, and cost-effective treatment options to address the global mental health treatment gap. The rapid onset of effects observed in several studies supports implementing structured, time-limited digital protocols. A proposed four-phase implementation framework (technical infrastructure development, clinical validation pathways, accessible technology deployment, training, and ethical implementation) provides a roadmap for translating these findings into routine clinical practice.

Future research should prioritize: (a) longitudinal studies evaluating long-term effect maintenance with minimum 6–12 month follow-up periods; (b) direct comparisons between AI-based modalities; (c) neuroimaging biomarker studies to predict treatment response; (d) adequately powered replication trials in cognitive impairment populations; (e) cultural adaptation and validation in low- and middle-income countries; and (f) algorithmic fairness evaluation across demographic subgroups.

## Figures and Tables

**Table 1:** Summary characteristics of included studies by research question (k = 186).

Characteristic	RQ1 Exec. Function	RQ2 Emotion Reg.	RQ3 Mechanisms	RQ4 Mental Health	RQ5 Cog. Impair.	RQ6 Other Clin.
Number of studies (*k*)	12	16	41	94	11	12
Total participants (*n*)	1484	2452	4454	12,899	482	984
Study design, *n* (%)						
RCT	8 (66.7)	12 (75.0)	27 (65.9)	57 (60.6)	10 (90.9)	7 (58.3)
Cluster-RCT	0 (0.0)	1 (6.3)	0 (0.0)	3 (3.2)	0 (0.0)	0 (0.0)
Quasi-experimental	0 (0.0)	0 (0.0)	0 (0.0)	2 (2.1)	0 (0.0)	1 (8.3)
Pilot/Feasibility	1 (8.3)	2 (12.5)	2 (4.9)	5 (5.3)	0 (0.0)	3 (25.0)
Other designs ^a^	3 (25.0)	1 (6.3)	12 (29.3)	27 (28.7)	1 (9.1)	1 (8.3)
Delivery format, *n* (%)						
Web-based platforms	2 (16.7)	5 (31.3)	5 (12.2)	25 (26.6)	1 (9.1)	2 (16.7)
Mobile applications	0 (0.0)	5 (31.3)	2 (4.9)	15 (16.0)	0 (0.0)	0 (0.0)
Chatbot/Conversational AI	0 (0.0)	1 (6.3)	0 (0.0)	8 (8.5)	0 (0.0)	0 (0.0)
Neuromodulation ^b^	0 (0.0)	0 (0.0)	11 (26.8)	2 (2.1)	0 (0.0)	0 (0.0)
Virtual reality	0 (0.0)	0 (0.0)	0 (0.0)	1 (1.1)	3 (27.3)	1 (8.3)
Ecological momentary	1 (8.3)	2 (12.5)	0 (0.0)	5 (5.3)	0 (0.0)	0 (0.0)
Other digital modalities	9 (75.0)	3 (18.8)	23 (56.1)	38 (40.4)	7 (63.6)	9 (75.0)
Effect size (Hedges’ g)						
Pooled g [95% CI]	0.61 [0.44, 0.78]	0.61 [0.51, 0.70]	0.49 ^c^	0.72 [0.61, 0.83]	1.02 [0.71, 1.33]	0.19 [0.02, 0.36]
I^2^ (%)	68.2	17.5	82.1	76.3	58.7	45.2
95% Prediction interval ^d^	[0.12, 1.10]	[0.41, 0.80]	[−0.18, 1.16]	[0.15, 1.29]	[0.38, 1.66]	[−0.08, 0.46]
Publication years	2020–2025	2021–2025	2020–2025	2020–2025	2021–2025	2020–2025
Risk of bias, *n* (%)						
Low	5 (41.7)	7 (43.8)	17 (41.5)	39 (41.5)	5 (45.5)	5 (41.7)
Some concerns	5 (41.7)	6 (37.5)	15 (36.6)	36 (38.3)	4 (36.4)	5 (41.7)
High	2 (16.7)	3 (18.8)	9 (22.0)	19 (20.2)	2 (18.2)	2 (16.7)

Note. RQ1 = AI-delivered interventions targeting executive function; RQ2 = AI-delivered interventions targeting emotion regulation; RQ3 = Neuropsychological mechanisms; RQ4 = Mental health clinical subgroup; RQ5 = Cognitive impairment clinical subgroup; RQ6 = Other clinical populations. k = number of studies; *n* = total participants; CI = confidence interval; RCT = randomized controlled trial. ^a^ Other designs include observational (*n* = 9), pre-post (*n* = 7), and unspecified designs (*n* = 29). ^b^ Neuromodulation includes tDCS and rTMS protocols targeting prefrontal regions. ^c^ Median effect size reported for RQ3 due to heterogeneity of mechanism measures. ^d^ 95% prediction intervals indicate the expected range of true effects in future similar studies, accounting for between-study heterogeneity; intervals containing zero indicate that some future studies may show null or negative effects even if the pooled estimate is positive.

**Table 2:** Summary of Included RQ2 Studies by Emotion Regulation Domain.

ER Domain	k	*n*	*g* [Range or Value]
Mindfulness-based ER programs	2	112	1.02–1.11
AI-platform/transdiagnostic digital ER	4	1022	0.56–0.82
Compassion-focused/CFT online ER	2	271	0.70–0.79
Cognitive reappraisal/CBM app	2	206	0.65
App-based mindfulness for healthcare/clinical	2	172	0.45–0.50
Internet-delivered anger/NSSI ER	2	434	0.27–0.56
AI emotion detection/EFL/chatbot	3	235	0.40–0.56

Note. ER = emotion regulation; *k* = number of studies; *g* = Hedges’ *g*. Pooled subgroup estimates by domain are not reported, as the number of studies per category is insufficient for stable pooling. Individual effect sizes derived from reported *d*, standardized coefficients, or partial η^2^, converted to Hedges’ *g* via standard formulae.

**Table 3:** Sensitivity analyses results.

Analysis	k	g	95% CI	Conclusion
All studies	186	0.68	[0.58, 0.78]	Reference
RCTs only	121	0.71	[0.60, 0.82]	Robust
Low RoB only	78	0.74	[0.61, 0.87]	Effect maintained
Trim-and-fill adjusted	186 + 8	0.62	[0.52, 0.72]	Effect robust

Note. k = number of studies; g = Hedges’ g; CI = confidence interval; RCT = randomized controlled trial; RoB = risk of bias.

## Data Availability

No new data were created or analyzed in this study.

## Supplementary Materials

The following supporting information can be downloaded at: https://www.mdpi.com/article/10.3390/brainsci16040389/s1, Table S1: PRISMA 2020 Checklist; Table S2: Characteristics of studies included in the meta-analysis (k = 186); Table S3: Meta-regression results for moderator analyses; Table S4: GRADE evidence certainty assessment for primary outcomes.

## Abbreviations

The following abbreviations are used in this manuscript:

Clinical/Psychological Terms	
ACT	Acceptance and Commitment Therapy
ADHD	Attention Deficit Hyperactivity Disorder
ASD	Autism Spectrum Disorder
CBT	Cognitive Behavioral Therapy
DMN	Default Mode Network
EF	Executive Function
ER	Emotion Regulation
MCI	Mild Cognitive Impairment
PTSD	Post-Traumatic Stress Disorder
WM	Working Memory
Neuroimaging/Physiological Terms	
DLPFC	Dorsolateral Prefrontal Cortex
ECG	Electrocardiogram
EEG	Electroencephalography
ERP/ERPs	Event-Related Potential(s)
fMRI	Functional Magnetic Resonance Imaging
HRV	Heart Rate Variability
LPP	Late Positive Potential
PFC	Prefrontal Cortex
SCRs	Skin Conductance Responses
tDCS	Transcranial Direct Current Stimulation
Technology/Intervention Terms	
AI	Artificial Intelligence
EMI	Ecological Momentary Intervention
PA	Physical Activity
VR	Virtual Reality
Methodological/Statistical Terms	
CI	Confidence Interval
g	Hedges’ g (standardized mean difference)
I^2^	I-squared (heterogeneity statistic)
k	Number of studies
*n*	Total Sample Size
NOS	Newcastle-Ottawa Scale
PRISMA	Preferred Reporting Items for Systematic Reviews and Meta-Analyses
RCT/RCTs	Randomized Controlled Trial(s)
RoB 2	Risk of Bias Tool 2.0
ROBINS-I	Risk of Bias in Non-Randomized Studies of Interventions
RQ/RQs	Research Question(s)
