# Supplementary material for: Neuropsychological Mechanisms Associated with the Effectiveness of AI-Delivered Health Promotion Programs: A Comprehensive Meta-Analysis
Source: Brain Sci. 2026 Mar 31;16(4):389. doi: 10.3390/brainsci16040389 (PMC13114729; doi:10.3390/brainsci16040389)
Supplement: Supplementary file 1 [file brainsci-16-00389-s001.zip › Table_S1_PRISMA_2020_checklist.pdf]

# PRISMA 2020 Checklist

| Section and Topic | Item # | Checklist item | Location where item is reported |
|---|---|---|---|
| **TITLE Neuropsychological Mechanisms Associated with the Effectiveness of AI-Delivered Health Promotion Programs: A Comprehensive Meta-Analysis** | | | |
| Title | 1 | Identify the report as a systematic review. | Title page; line 1 ("Systematic Review") and full title |
| **ABSTRACT** | | | |
| Abstract | 2 | See the PRISMA 2020 for Abstracts checklist. | Abstract section (structured abstract with Background, Methods, Results, Conclusions) |
| **INTRODUCTION** | | | |
| Rationale | 3 | Describe the rationale for the review in the context of existing knowledge. | Section 1.1–1.8 (Background and Rationale) |
| Objectives | 4 | Provide an explicit statement of the objective(s) or question(s) the review addresses. | Section 1.9 (Research Questions: RQ1–RQ6 explicitly stated) |
| **METHODS** | | | |
| Eligibility criteria | 5 | Specify the inclusion and exclusion criteria for the review and how studies were grouped for the syntheses. | Section 2.2 (Eligibility Criteria: 2.2.1 Inclusion Criteria; 2.2.2 Exclusion Criteria) |
| Information sources | 6 | Specify all databases, registers, websites, organisations, reference lists and other sources searched or consulted to identify studies. Specify the date when each source was last searched or consulted. | Section 2.3 (Information Sources and Search Strategy); databases: PubMed/MEDLINE, PsycINFO, Embase, Web of Science, Cochrane CENTRAL, IEEE Xplore; timeframe: 2020–2025 |
| Search strategy | 7 | Present the full search strategies for all databases, registers and websites, including any filters and limits used. | Section 2.3 (search terms for AI/digital interventions, executive function, emotion regulation, and health promotion provided) |
| Selection process | 8 | Specify the methods used to decide whether a study met the inclusion criteria of the review, including how many reviewers screened each record and each report retrieved, whether they worked independently, and if applicable, details of automation tools used in the process. | Section 2.4 (Study Selection and Data Extraction); Section 3.1 (two independent reviewers, disagreements resolved via discussion or third reviewer) |
| Data collection process | 9 | Specify the methods used to collect data from reports, including how many reviewers collected data from each report, whether they worked independently, any processes for obtaining or confirming data from study investigators, and if applicable, details of automation tools used in the process. | Section 2.4 (standardized data extraction form; corresponding authors contacted for missing data) |
| Data items | 10a | List and define all outcomes for which data were sought. Specify whether all results that were compatible with each outcome domain in each study were sought (e.g. for all measures, time points, analyses), and if not, the methods used to decide which results to collect. | Section 2.2.1 (Inclusion Criteria: executive function outcomes, emotion regulation outcomes); Table 1 (outcome categories by RQ) |
| | 10b | List and define all other variables for which data were sought (e.g. participant and intervention characteristics, funding sources). Describe any assumptions made about any missing or unclear information. | Section 2.4 (study characteristics, participant demographics, intervention details); Table 1 (study design, delivery format, publication years) |
| Study risk of bias assessment | 11 | Specify the methods used to assess risk of bias in the included studies, including details of the tool(s) used, how many reviewers assessed each study and whether they worked independently, and if applicable, details of automation tools used in the process. | Section 2.5 (Cochrane RoB 2 for RCTs; ROBINS-I for non-randomized studies; two independent reviewers) |
| Effect measures | 12 | Specify for each outcome the effect measure(s) (e.g. risk ratio, mean difference) used in the synthesis or presentation of results. | Section 2.6 (Hedges' g with 95% CI for standardized mean differences) |
| Synthesis methods | 13a | Describe the processes used to decide which studies were eligible for each synthesis (e.g. tabulating the study intervention characteristics and comparing against the planned groups for each synthesis (item #5)). | Section 2.6; Section 3.1 (studies distributed across six RQs based on primary outcomes and population characteristics) |

PRISMA

# PRISMA 2020 Checklist

| Section and Topic | Item # | Checklist item | Location where item is reported |
|---|---|---|---|
| | 13b | Describe any methods required to prepare the data for presentation or synthesis, such as handling of missing summary statistics, or data conversions. | Section 2.6 (effect sizes calculated as Hedges' g to account for small sample sizes) |
| | 13c | Describe any methods used to tabulate or visually display results of individual studies and syntheses. | Section 2.6; Figures 2–9 (forest plots, bar charts, funnel plots); Tables 1–3 |
| | 13d | Describe any methods used to synthesize results and provide a rationale for the choice(s). If meta-analysis was performed, describe the model(s), method(s) to identify the presence and extent of statistical heterogeneity, and software package(s) used. | Section 2.6 (random-effects models; Q statistic and $I^2$ for heterogeneity; R software with metafor package) |
| | 13e | Describe any methods used to explore possible causes of heterogeneity among study results (e.g. subgroup analysis, meta-regression). | Section 2.6 (subgroup analyses by RQ, moderator analyses by intervention type and population) |
| | 13f | Describe any sensitivity analyses conducted to assess robustness of the synthesized results. | Section 2.6 (sensitivity analyses); Section 3.9 (leave-one-out, RCT-only, low RoB-only analyses); Table 3 |
| Reporting bias assessment | 14 | Describe any methods used to assess risk of bias due to missing results in a synthesis (arising from reporting biases). | Section 2.6 (funnel plots, Egger's regression test, trim-and-fill method) |
| Certainty assessment | 15 | Describe any methods used to assess certainty (or confidence) in the body of evidence for an outcome. | Section 2.6 (GRADE described); Section 3.9.2; Table S4 (GRADE for RQ1–RQ6) |
| **RESULTS** | | | |
| Study selection | 16a | Describe the results of the search and selection process, from the number of records identified in the search to the number of studies included in the review, ideally using a flow diagram. | Section 3.1 (1,247 records → 412 duplicates removed → 888 screened → 300 full-text assessed → 186 included); Figure 1 (PRISMA flow diagram) |
| | 16b | Cite studies that might appear to meet the inclusion criteria, but which were excluded, and explain why they were excluded. | Section 3.1 (114 articles excluded: 57 for no AI component, 56 for no EF/ER outcomes, 1 for insufficient data) |
| Study characteristics | 17 | Cite each included study and present its characteristics. | Table 1 (summary by RQ); Supplementary Materials Table S1 (full characteristics of k=186 studies); References [101–286] |
| Risk of bias in studies | 18 | Present assessments of risk of bias for each included study. | Section 3.8; Table 1 (risk of bias by RQ); Figure 8 (risk of bias summary) |
| Results of individual studies | 19 | For all outcomes, present, for each study: (a) summary statistics for each group (where appropriate) and (b) an effect estimate and its precision (e.g. confidence/credible interval), ideally using structured tables or plots. | Sections 3.2–3.7; Figures 2–7 (forest plots with individual study effects and 95% CIs); Tables 1–2 |
| Results of syntheses | 20a | For each synthesis, briefly summarise the characteristics and risk of bias among contributing studies. | Table 1 (characteristics and RoB by RQ); Section 3.8 (risk of bias summary) |
| | 20b | Present results of all statistical syntheses conducted. If meta-analysis was done, present for each the summary estimate and its precision (e.g. confidence/credible interval) and measures of statistical heterogeneity. If comparing groups, describe the direction of the effect. | Sections 3.2–3.7 (pooled g with 95% CI; $I^2$ for each RQ); Table 1 (overall g=0.68 [0.58, 0.78]; $I^2$=73.4%) |
| | 20c | Present results of all investigations of possible causes of heterogeneity among study results. | Sections 3.2.2, 3.3.1, 3.4.1–3.4.3, 3.5.2 (moderator analyses by intervention modality, subdomain, delivery format) |
| | 20d | Present results of all sensitivity analyses conducted to assess the robustness of the synthesized results. | Section 3.9 (Sensitivity Analyses); Table 3 (RCTs only g=0.71; low RoB only g=0.74; trim-and-fill g=0.62) |
| Reporting biases | 21 | Present assessments of risk of bias due to missing results (arising from reporting biases) for each synthesis assessed. | Section 3.8 (funnel plot asymmetry for RQ3/RQ4; Egger's test z=2.14, p=.032 for mental health; trim-and-fill adjusted g=0.65); |

PRISMA

# PRISMA 2020 Checklist

| Section and Topic | Item # | Checklist item | Location where item is reported |
|---|---|---|---|
| | | | Figure 9 (funnel plots) |
| Certainty of evidence | 22 | Present assessments of certainty (or confidence) in the body of evidence for each outcome assessed. | Formally assessed using GRADE for all six outcomes; MODERATE for RQ1–RQ4 and RQ6; LOW for RQ5; Section 3.9.2 and Table S4 |
| **DISCUSSION** | | | |
| Discussion | 23a | Provide a general interpretation of the results in the context of other evidence. | Section 4 (Discussion: Sections 4.1–4.4 interpret findings in context of prior literature); Section 4.8 (Comparative Analysis) |
| | 23b | Discuss any limitations of the evidence included in the review. | Section 4.6 (Technical and Methodological Considerations: heterogeneity, blinding challenges, publication bias) |
| | 23c | Discuss any limitations of the review processes used. | Section 4.7 (Limitations and Future Directions: heterogeneity of interventions, short follow-up, limited diversity, implementation barriers) |
| | 23d | Discuss implications of the results for practice, policy, and future research. | Section 4.5 (Clinical Decision-Making Implications); Section 4.9 (Implementation Framework); Section 5 (Conclusions); Figure 10 |
| **OTHER INFORMATION** | | | |
| Registration and protocol | 24a | Provide registration information for the review, including register name and registration number, or state that the review was not registered. | Section 2.1 (Open Science Framework; osf.io/aunks; DOI 10.17605/OSF.IO/AUNKS) |
| | 24b | Indicate where the review protocol can be accessed, or state that a protocol was not prepared. | Section 2.1 (protocol pre-registered with OSF) |
| | 24c | Describe and explain any amendments to information provided at registration or in the protocol. | Not reported; no amendments mentioned |
| Support | 25 | Describe sources of financial or non-financial support for the review, and the role of the funders or sponsors in the review. | Funding statement: "This research received no external funding" |
| Competing interests | 26 | Declare any competing interests of review authors. | Conflicts of Interest statement: "The authors declare no conflicts of interest" |
| Availability of data, code and other materials | 27 | Report which of the following are publicly available and where they can be found: template data collection forms; data extracted from included studies; data used for all analyses; analytic code; any other materials used in the review. | Data Availability Statement: "No new data were created or analyzed in this study"; Supplementary Materials (Table S1, Table S2) |

*From:* Page MJ, McKenzie JE, Bossuyt PM, Boutron I, Hoffmann TC, Mulrow CD, et al. The PRISMA 2020 statement: an updated guideline for reporting systematic reviews. BMJ 2021;372:n71. doi: 10.1136/bmj.n71.
This work is licensed under CC BY 4.0. To view a copy of this license, visit https://creativecommons.org/licenses/by/4.0/
