# Supplementary material for: Neuropsychological Mechanisms Associated with the Effectiveness of AI-Delivered Health Promotion Programs: A Comprehensive Meta-Analysis
Source: Brain Sci. 2026 Mar 31;16(4):389. doi: 10.3390/brainsci16040389 (PMC13114729; doi:10.3390/brainsci16040389)
Supplement: Supplementary file 1 [file brainsci-16-00389-s001.zip › Table_S3_Meta_Regression_Results.pdf]

. Meta-Regression Results for Moderator Analyses

Variable	k	β	95% CI	SE	p	R² (%)	Interpretation
**s Moderators**							
year (2020-2025)	186	0.03	[-0.02, 0.08]	0.025	.23	1.2%	NS; no temporal trend
(log-transformed)	186	-0.08	[-0.15, -0.01]	0.036	.028	3.8%	Small-study effects present
duration (weeks)	178	0.01	[-0.01, 0.03]	0.010	.34	0.8%	NS; duration not moderating
luration (months)	47	-0.02	[-0.04, -0.003]	0.009	.04	8.2%	Effect attenuation over time
mptom severity	42	0.12	[0.02, 0.22]	0.051	.02	11.4%	Higher severity → larger effects
ipant age (years)	186	0.002	[-0.004, 0.008]	0.003	.54	0.3%	NS; age not moderating
**l Moderators (Q-test for subgroup differences)**							
dition type	179	—	—	—	.008	Q=7.02	Active controls: smaller effects
ntrol	72	g=0.52	[0.40, 0.64]	—	—	—	
no treatment	107	g=0.78	[0.66, 0.90]	—	—	—	
rating	186	—	—	—	.18	Q=3.41	NS; RoB not moderating
ype	186	—	—	—	.03	Q=4.87	Clinical > non-clinical
opulations	117	g=0.73	[0.62, 0.84]	—	—	—	
ical samples	69	g=0.58	[0.44, 0.72]	—	—	—	
ation tier	186	—	—	—	.12	Q=4.24	NS; tier not moderating
region	186	—	—	—	.24	Q=4.21	NS; region not moderating

andardized regression coefficient; CI = confidence interval; SE = standard error; R² = proportion of between-study variance explained; Q = Q-statistic for subgroup diff
Analyses conducted using random-effects meta-regression with restricted maximum likelihood estimation and robust variance estimation (clubSandwich package, CR2 c
l moderators, effect sizes (g) are reported for each subgroup level.
