# Supplementary material for: Neuropsychological Mechanisms Associated with the Effectiveness of AI-Delivered Health Promotion Programs: A Comprehensive Meta-Analysis
Source: Brain Sci. 2026 Mar 31;16(4):389. doi: 10.3390/brainsci16040389 (PMC13114729; doi:10.3390/brainsci16040389)
Supplement: Supplementary file 1 [file brainsci-16-00389-s001.zip › Table_S4_GRADE_Assessment.pdf]

. GRADE Evidence Certainty Assessment for Primary Outcomes

	k	Risk of Bias	Inconsistency	Indirectness	Imprecision	Pub. Bias	Effect (g)	95% CI	C
ive Function	12	No serious	Serious [a]	No serious	No serious	None	0.61	[0.44, 0.78]	**MO**
n Regulation	16	No serious	No serious	Serious [b]	No serious	None	0.61	[0.51, 0.70]	**MO**
nisms	41	No serious	V. Serious [c]	No serious	No serious	None	0.49	[0.35, 0.63]	**MO**
Health	94	No serious	Serious [d]	No serious	No serious	Suspected [e]	0.72	[0.61, 0.83]	**MO**
ve Impairment	11	No serious	No serious	No serious	Serious [f]	Suspected [g]	1.02	[0.71, 1.33]	
Clinical	12	No serious	No serious	Serious [h]	No serious	None	0.19	[0.02, 0.36]	**MO**

dicating substantial heterogeneity across executive function measures and intervention types.

three of the 16 included studies ([113], [114], [116]) relied on approximate or converted effect sizes, introducing potential imprecision in effect estimation; [114] is char
thors as a rule-based rather than AI-adaptive chatbot. Heterogeneity was low-to-moderate (I^2 = 17.5%), which does not warrant a downgrade for inconsistency.

dicating very high heterogeneity due to diversity of mechanistic measures (neural, physiological, cognitive).

dicating substantial heterogeneity across mental health conditions and intervention approaches.

= 0.032 suggesting potential publication bias; trim-and-fill adjusted g = 0.65.

tive sample (N = 482; confirmed AI-delivered pool k = 7, N ≈ 280; four studies excluded from primary estimate: [265] secondary meta-analysis, [268] aerobic exercise w
gramme without AI, [272] teacher-delivered without AI) and wide confidence interval indicating imprecision; I^2 = 58.7% reflects moderate between-study variance in the

= 0.08 suggesting potential small-study effects; trim-and-fill adjusted g = 0.85.

us population category encompassing diverse clinical conditions (chronic pain, substance use, rehabilitation); four of 12 assigned studies were protocol papers, a Delphi
I interventions (refs [277], [279], [284], [286]); confirmed evidence base k = 8 (I^2 = 45.2%).

nty ratings: HIGH = further research very unlikely to change confidence; MODERATE = further research likely to impact confidence; LOW = further research very likel
Y LOW = estimate uncertain. Starting from HIGH for RCT evidence, downgraded by one domain for each serious concern.
